# Characterisation of C101248: A novel selective THIK-1 channel inhibitor for the modulation of microglial NLRP3-inflammasome

**DOI:** 10.1016/j.neuropharm.2022.109330

**Published:** 2023-02-15

**Authors:** Bernardino Ossola, Ali Rifat, Anna Rowland, Helen Hunter, Samuel Drinkall, Clare Bender, Mayida Hamlischer, Martin Teall, Russell Burley, Daneil F. Barker, David Cadwalladr, Louise Dickson, Jason M.K. Lawrence, Jenna R.M. Harvey, Marina Lizio, Xiao Xu, Edel Kavanagh, Toni Cheung, Steve Sheardown, Catherine B. Lawrence, Michael Harte, David Brough, Christian Madry, Kim Matthews, Kevin Doyle, Keith Page, Justin Powell, Nicola L. Brice, Roland W. Bürli, Mark B. Carlton, Lee A. Dawson

**Affiliations:** a Cerevance Ltd, 418 Cambridge Science Park, Milton Road, Cambridge, CB4 0PZ, UK; b Charité – Universitätsmedizin Berlin, corporate member of Freie Universität Berlin and Humboldt-Universität zu Berlin, Institute of Neurophysiology, Charitéplatz 1, 10117 Berlin, Germany; c Berlin Institute of Health at Charité – Universitätsmedizin Berlin, Charitéplatz 1, 10117, Berlin, Germany; d Division of Pharmacy & Optometry, School of Health Sciences, Faculty of Biology, Medicine and Health, University of Manchester, Manchester Academic Health Science Centre, Manchester, UK; e Division of Neuroscience and Experimental Psychology, School of Biological Sciences, Faculty of Biology, Medicine and Health, University of Manchester, Manchester Academic Health Science Centre, Manchester, UK; f Geoffrey Jefferson Brain Research Centre, The Manchester Academic Health Science Centre, Northern Care Alliance NHS Group, University of Manchester, Manchester, UK; g The Lydia Becker Institute of Immunology and Inflammation, University of Manchester, Manchester, UK

**Keywords:** THIK-1, KCNK13, NLRP3 inflammasome, Microglia, Neuroinflammation, Alzheimer's disease, NETSseq, Nuclear Enriched Transcript Sort sequencing, THIK-1, tandem pore domain halothane-inhibited K^+^ channel 1, KCNK13, potassium two pore domain channel subfamily K member 13 gene, K2P, K^+^ two pore domain channel, NLRP3, NLR Family Pyrin Domain Containing 3, AD, Alzheimer's disease, PAMPs, pathogen-associated molecular patterns, DAMPs, damage-associated molecular patterns, IL-1β, Interleukin 1 beta

## Abstract

Neuroinflammation, specifically the NLRP3 inflammasome cascade, is a common underlying pathological feature of many neurodegenerative diseases. Evidence suggests that NLRP3 activation involves changes in intracellular K^+^. Nuclear Enriched Transcript Sort Sequencing (NETSseq), which allows for deep sequencing of purified cell types from human post-mortem brain tissue, demonstrated a highly specific expression of the tandem pore domain halothane-inhibited K^+^ channel 1 (THIK-1) in microglia compared to other glial and neuronal cell types in the human brain. NETSseq also showed a significant increase of THIK-1 in microglia isolated from cortical regions of brains with Alzheimer's disease (AD) relative to control donors.

Herein, we report the discovery and pharmacological characterisation of C101248, the first selective small-molecule inhibitor of THIK-1. C101248 showed a concentration-dependent inhibition of both mouse and human THIK-1 (IC50: ∼50 nM) and was inactive against K2P family members TREK-1 and TWIK-2, and Kv2.1. Whole-cell patch-clamp recordings of microglia from mouse hippocampal slices showed that C101248 potently blocked both tonic and ATP-evoked THIK-1 K^+^ currents. Notably, C101248 had no effect on other constitutively active resting conductance in slices from THIK-1-depleted mice. In isolated microglia, C101248 prevented NLRP3-dependent release of IL-1β, an effect not seen in THIK-1-depleted microglia.

In conclusion, we demonstrated that inhibiting THIK-1 (a microglia specific gene that is upregulated in brains from donors with AD) using a novel selective modulator attenuates the NLRP3-dependent release of IL-1β from microglia, which suggests that this channel may be a potential therapeutic target for the modulation of neuroinflammation in AD.

## Highlights

• THIK-1 mRNA and protein are highly enriched in microglia in human cortical tissue.
• THIK-1 mRNA is increased in microglial from AD brains compared to non-AD controls.
• Disclosure of C101248, the first selective small-molecule to block THIK-1 currents.
• THIK-1 inhibition prevented the NLRP3-dependent release of IL-1β from microglia.

## 1 Introduction

Neuroinflammation is a common underlying pathological feature of most neurological disorders. Chronic neuroinflammation is evident in most, if not all, progressive neurodegenerative disorders such as Alzheimer's disease (AD), amyotrophic lateral sclerosis (ALS), Parkinson's disease (PD) (Heneka et al., 2014; Thonhoff et al., 2018), and autoimmune disorders such as multiple sclerosis (Barclay and Shinohara, 2017). It can also mediate ongoing damage to the central nervous system (CNS) following brain injuries such as stroke (Jayaraj et al., 2019) or traumatic brain injury (Simon et al., 2017). The importance of neuroinflammation in disease is further underlined by findings that variants of genes for immune receptors, such as TREM2 and CD33 are risk factors for, and afford selective vulnerability to, a variety of neurodegenerative diseases, including AD and PD (Jay et al., 2017; Li and Zhang, 2021; Li et al., 2021). Many of these genes are exclusively expressed in microglia indicating a key role of this cell type in neuroinflammation and pathogenic disease processes (Colonna and Butovsky, 2017; Ransohoff, 2016).

Microglia are considered the brain's resident macrophages and play a central role in the development, homeostasis, and ultimately, diseases of the CNS. Adult microglia have a sentinel type role of surveying their environment and interacting with essentially all CNS components. As such, they have a marked impact on normal brain function and maintenance of tissue integrity. To achieve this, microglia rapidly adapt to their environment by increasing their cell number, modifying their function and activation states (of which they have a broad spectrum), and mediating and responding to damage, infection, and inflammation. Specifically, during these environmental challenges, microglia change their morphology, from the ramified sentinel phenotype to more of an amoeboid constitution. This is accompanied by higher levels of phagocytic activity, increased proliferation, and a cascade of cellular biochemistry, resulting in cytokine release and an orchestrated inflammatory response to resolve the adverse event or challenge (Li and Barres, 2018). Activation of microglia is a pathological hallmark of all neurodegenerative diseases and can alter disease processes and progression. Although an initially favourable response to environmental changes, there is clear evidence that microglial activation becomes dysfunctional and progresses the neurodegenerative disease process. Although the biochemical processes involved are complex, there are several pathways identified as critical to the disease aetiology and therefore potential interventional points for therapeutic approaches; one such process is that involving the nod-like receptor family pyrin domain containing 3 (NLRP3) cascades (Heneka et al., 2018).

NLRP3 is a component of the innate immune system that functions as a pattern recognition receptor (PRR). It identifies pathogen-associated molecular patterns (PAMPs) and damage-associated molecular patterns (DAMPs) which then triggers downstream inflammatory pathways to eliminate microbial infection and repair damaged tissues (Kelley et al., 2019). Canonical activation of the NLRP3 inflammasome requires a two-step process, priming and activation. The first step, priming, usually occurs through the stimulation of toll-like receptors (TLRs) (Qiao et al., 2012; Toma et al., 2010) which mediates upregulation of the nuclear factor-kappa B pathway to increase the expression of NLRP3 and prointerleukin-1β (pro-IL-1β). The second step, activation, triggers the formation of the inflammasome complex of NLRP3 with the adaptor ASC protein and caspase-1. This activated NLRP3 inflammasome leads to activation of caspase-1 which in turn induces the maturation of the proinflammatory cytokines, IL-1β and IL-18. The NLRP3 inflammasome is triggered by changes in intracellular potassium (K^+^) and K^+^ eﬄux alone can activate NLRP3, while high extracellular K^+^ blocks the activation of the NLRP3 inﬂammasome while avoiding other inﬂammasomes (Muñoz-Planillo et al., 2013; Pétrilli et al., 2007). Thus, a decrease in intracellular K^+^ is considered a trigger for NLRP3 inﬂammasome activation.

The role of K^+^ efflux in canonical NLRP3 activation in response to many stimuli is well documented and several ion channels are suggested to mediate this K^+^ current in microglia. One such channel is encoded by KCNK13 (K_2P_13.1) or K^+^ two pore domain channel subfamily K member 13 gene, which translates to a two-pore forming domain potassium channel known as tandem pore domain halothane-inhibited K^+^ channel 1 or THIK-1. THIK-1 together with THIK-2 (KCNK12) are members of the leak or background K^+^ channels (K_2P_) first cloned by Rajan et al. (2001). Electrophysiological studies show that THIK-1 is an outwardly rectifying channel with a very small single-channel conductance (∼5 pS at +100 mV) and short open time duration (<0.5 ms) (Kang et al., 2014). THIK-1 controls key microglial function across the spectrum including surveillance, ramification, phagocytosis and membrane voltage (Izquierdo et al., 2021; Madry et al., 2018a, 2018b). Importantly, blockade of THIK-1 also suppresses NLRP3-dependent release of proinflammatory IL-1β in the rodent brain (Madry et al., 2018b) and in cultured macrophages and microglia (Drinkall et al., 2022). In line with this, an increase in THIK-1 current density in microglia lacking P2Y13 receptor expression correlated with significantly upregulated IL-1ß levels (Kyrargyri et al., 2020). Thus, specific pharmacological targeting of THIK-1, which has been precluded so far due to the lack of selective compounds, may likely provide a therapeutic strategy of attenuating NLRP3-mediated proinflammatory processes that underlie many CNS diseases. In support of this hypothesis, a recent study comparing the whole genome sequence of 507 cases of AD with close relatives affected by AD and 4917 cognitive healthy controls, identified a novel SNP in KCNK13 as one of the risk genes (Zhang et al., 2019). It should be noted that the evidence for association of THIK-1 and AD was reduced following adjustment for age and sex (Zhang et al., 2019).

Using NETSseq (Xu et al., 2018) and histological methods, we show the microglial specific expression of THIK-1 in human post-mortem brain tissue from both non-demented controls and AD donors. Additionally, THIK-1 expression was upregulated in microglia from various cortical regions of AD donors compared to aged matched non-demented control brains.

Furthermore, we report the discovery and pharmacological characterisation of C101248, the first selective small-molecule inhibitor of THIK-1 currents. Our data demonstrate the neurophysiological characteristics of C101248 to inhibit human and rodent THIK-1 activity and the impact of this on activation of NLRP3 and subsequent IL-1β release. We therefore suggest that selective blockers of THIK-1 could reduce microglial NLRP3 inflammasome mediated inflammation and thereby have therapeutic utility in many of the NLRP3 related neuroinflammatory brain diseases.

## 2 Materials & methods

### 2.1 Compounds and reagents

The preparation of C101248, C101505 and ^3^H–C101505 are described in the Supporting Information. All other reagents were from Merck unless otherwise stated.

### 2.2 Animals

KCNK13 (THIK-1) knockout (KO) mice were obtained from MRC Harwell and maintained as homozygotes on a C57Bl/6 background. For the electrophysiology experiments on hippocampal slices Cx3Cr1^GFP^ knock-in/knock-out mice (Jung et al., 2000) were used, which express enhanced green fluorescent protein (EGFP) under the control of the endogenous Cx3Cr1 locus in brain microglia. Cx3Cr1^GFP^ mice were crossbred with THIK-1 KO mice to generate Cx3Cr1^GFP^/THIK-1 KO mice lacking THIK-1 expression constitutively. Housing of mice was in individually ventilated cages and maintained on a 12-h light/dark cycle and had access to ab libitum food and water. For all experiments either sex was used. All experimental procedures were performed under Home Office UK project licence in accordance with the Animals (Scientific Procedures) Act UK 1986 and approved by the University of Cambridge Animal Welfare and Ethical Review Body. All procedures involving handling of Cx3Cr1^GFP^/THIK-1 KO mice were performed in accordance with the German animal protection law and were approved by the local offices for health and social services. All THIK-1 KO mice were genotyped using the following Taqman MGB probes (ThermoFisher) and primers (Sigma): MmKCNK13 SNP Genotyping FP = 5′-GGTCGGCAGAGCACATCCT-3′ RP = 5′-CTGCAACTCCTGCGCTAGCT-3’; MmKCNK13 WT SNP Genotyping Probe = FAM-5′-CACCTGAACGAGGAC-3′-MGB; MmKCNK13 KO SNP Genotyping Probe = VIC-5′-CACCTGAATCGAGGAC-3′-MGB. Lysates were prepared from ear snips using Extract-N-Amp Tissue PCR Kit (Sigma). qPCR was run in 384-well plates with the following thermocycler conditions: 60 °C × 30 s, 95 °C × 10 min, then 40 cycles at 95 °C × 15 s, 60 °C × 1 min, lastly one cycle at 60 °C × 30 s.

### 2.3 Pharmacokinetic measurements

In CNS PK studies, rodents (C57BL/6 mice or Sprague Dawley rats) were administered C101248 by oral gavage at 10 or 30 mg/kg. At specified timepoints post dose (0.5, 1, 2 or 4 h), blood samples were collected and processed to plasma (centrifugation at 5000 rpm, 4 °C for 10 min). Immediately following blood withdrawal, cerebrospinal fluid (CSF) samples were collected from the cisterna magna, whole body perfusion was conducted, and brain samples were collected and weighed. Samples were stored frozen at −80 ± 10 °C until analysis. Plasma, CSF, and brain samples were analysed using a fit for purpose bioanalytical method (LC-MS/MS) against a calibration curve containing QC samples. Brain:plasma and CSF:plasma were calculated based on brain concentration/plasma concentration at the same timepoint. Unbound partition coefficient (Kp_u,u_) values were calculated based on unbound brain concentration/unbound plasma concentration at the same timepoint. Unbound concentrations were derived by adjusting plasma concentrations for the fraction unbound in plasma based on in vitro plasma protein binging (PPB) values and brain concentrations for the unbound fraction in brain tissue based on in vitro brain tissue binding (BTB) values.

### 2.4 HEK293 and CHO cells

HEK-293 cells were transfected with human THIK-1, TREK-1, TWIK-2, or mouse THIK-1 cDNA cloned into pcDNA3.1, using either Fugene HD transfection reagent (Promega) or electroporation (MaxCyte STX instrument). Cells stably expressing the relevant genes were selected based on resistance to 500 μg/ml geneticin (Gibco). For human THIK-1 and TWIK-2 expressing cells, monoclonal lines were isolated by limited dilution cloning; mouse THIK-1 and human TREK-1 expressing cells were maintained as polyclonal pools. The HEK-293 pools and monoclones stably expressing the relevant genes were maintained in DMEM (Life Technologies), supplemented with 10% FCIII (HyClone, Fetal clone III) and 500 μg/ml geneticin. A CHO-Kv2.1 cell line was purchased from Millipore (accession NM_004975) and maintained in F12 (Life Technologies), supplemented with 10% FCSIII (Thermo Scientific) and 500 μg/ml geneticin (Life Technologies). For thallium flux assays, banks of frozen cell aliquots were prepared the day prior to assay, cells were thawed into media and seeded into PDL coated 384-well plates (Corning) at 25,000 (human/mouse THIK-1, TREK-1) or 30,000 (TWIK-2) cells/well (25 μl/well). CHO-Kv2.1 cells were seeded into non-PDL coated plates (Corning) at 5000 cells/well. Plated cells were incubated overnight at 37 °C, 5% CO2 in a humidified atmosphere. For Q-Patch assays, cells were cultured for 7 days prior to assay, dissociated, and diluted to 1–2.5 million/ml in EX-CELL ACF serum free media containing HEPES (25 mM; Life Technologies), penicillin/streptomycin (100 U/ml, 100 μg/ml; Life Technologies) and a trypsin inhibitor (0.04 mg/ml).

#### 2.4.1 THP1 cells

THP1 cells, obtained from ATCC, are monocytes isolated from peripheral blood of a leukaemia patient. THP1 cells were grown in RPMI 1640 supplemented with 10% fetal bovine serum (FBS, Hyclone), 1% PenStrep (Invitrogen), and 1% GlutaMax (Invitrogen), and 0.05 mM of 2-mercaptoethanol. To overexpress human THIK-1, THP1 cells were differentiated with 100 ng/ml PMA for 24 h prior to transfection with THIK-1 mRNA synthesized as described in the supplementary material. Cell suspensions (2.5 × 10^6^ cells in 25 ml) in the presence of 200 μg/ml mRNA were electroporated with the MaxCyte (STX instrument) in an OC-25 MaxCyte Electroporation cassette using manufacturer settings for THP1 cells. Cell suspensions were transferred to a 6-well plate and allowed to recover for 20 min at 37 °C. Cells were seeded into 384-well plates (10,000 cells/well) for thallium flux assays or 96-well plates (50,000 cells/well) for inflammasome assays the following day. Overexpressing and mock-transfected cells were primed with 100 ng/ml of LPS for 3.5 h prior to addition of compounds 30 min before stimulation with 5 mM ATP. Supernatants were collected 2 h after addition of ATP and stored at −20 °C until the detection of IL-1β.

#### 2.4.2 Neonatal microglia

Neonatal microglia were isolated from C57BL/6 P1-4 mouse pups (Charles River, UK) as previously described (Carrillo-Jimenez et al., 2018). In brief, brains were dissected by removing meninges, cerebellum, and olfactory bulbs before tissue was finely minced with a scalpel then digested with papain solution for 20 min at 37 °C. Tissue was dissociated and the resulting cell suspension was passed through a 40 μm cell strainer (Greiner Bio-One). Cells were centrifuged at 300 g for 5 min then resuspended in culture medium (DMEM, 10% FBS, 1% PenStrep) and plated in T75 collagen-coated flasks (Greiner Bio-One). A full medium change was caried out on day 2 while a half medium was changed on day 5 in the presence of 5 ng/ml of mouse GM-CSF (R&D Systems). Microglia were shaken off between day 9 and 11 and plated onto poly-d-lysine coated 96-well plates (μclear, Greiner Bio-One) at a density of 20,000 cells/well. The following day cells were primed with LPS (100 ng/ml) for 3.5 h prior to treatment with C101248, MCC950 (Tocris), or vehicle (0.1% DMSO) for 30 min. NLRP3 was then activated by a complete medium change with an isotonic K^+^-free buffer (148 mM NaCl, 10 mM HEPES, 10 mM glucose, 2 mM CaCl_2_, 1 mM MgCl_2_ at pH 7.4) in the presence of test compounds or DMSO for 1 h after which supernatants were collected and stored at −20 °C until the measurement of IL-1β.

#### 2.4.3 Adult microglia

Microglia were isolated from C57BL/6 male and female mice aged 6–10 weeks (Charles River, UK) as previously described (Drinkall et al., 2022). In brief, brains were isolated, minced, and digested using a Neural Tissue Dissociation Kit (Miltenyi Biotec Ltd., UK) according to manufacturer's instructions. The resulting suspension was dissociated using a Dounce tissue grinder (Sigma). Myelin was removed by centrifuging cell suspension in 33% Percoll (GE Healthcare) for 10 min at 1000 g with low break and aspiration of myelin layer. Cells were pelleted by diluting 1:4 in Ca^2+^/Mg^2+^ free Hank's balanced salt solution (CMF-HBSS, Gibco) and centrifuged for 10 min at 500 g. Cells were incubated with anti-CD11b magnetic microbeads (Miltenyi Biotec Ltd., UK) in MACS buffer (PBS without Ca^2+^ and Mg^2+^ with 2 mM ethylenediaminetetraacetic acid and 0.5% bovine serum albumin) and sorted by positive selection with a LS column (Miltenyi Biotec Ltd., UK) according to manufacturer's instructions. The eluted microglia were counted and plated onto 96-well Cell + plates (Sarstedt) at a density of 30,000 cells per well in culture medium (DMEM/F12, 10% FBS, 1% PenStrep, 2 mM glutamine, 20 ng/ml IL-34 (R&D Systems), and 50 ng/ml transforming growth factors-*β*1 (Miltenyi Biotec Ltd., UK)). After 8 days in culture cells were primed with LPS (1 μg/ml, 4 h) then treated with C101248, MCC950, or vehicle (1% DMSO) in serum free media for 30 min. Following drug incubation NLRP3 was stimulated with ATP (5 mM, 1 h). Supernatants were collected and stored at −20 °C until the measurement of IL-1β.

### 2.5 Thallium influx assay

A fluorescence-based thallium influx assay (Molecular Devices potassium assay kit) was used to monitor channel activity. In brief, component A was reconstituted in 17 ml of chloride-free assay buffer (130 mM sodium d-gluconate, 5 mM potassium d-gluconate, 2 mM calcium d-gluconate, 1 mM magnesium d-gluconate, 5 mM NaHCO3, 20 mM HEPES; pH adjusted to 7.4 with NaOH and filtered (0.45 μm filter)) and component C reconstituted in 30 μl of DMSO. Components A and C were combined, and probenecid was added (Life Technologies; final assay concentration (FAC) 4.75 mM for HEK cell lines and 2.47 mM for CHO-Kv2.1) to provide the final dye solution. Media was removed from the cell plates and replaced with dye (20 μl/well) and the plates were incubated at room temperature in the dark for 60 min. On the day of the assay, a diluted compound plate (3x FAC in 1.5% DMSO) was created. Control wells contained either DMSO vehicle (1.5%, 3x FAC) or tetrapentylammonium chloride (TPA; Sigma, 258962; 1.5 mM in 1.5% DMSO, 3x FAC). A 3x thallium stimulation plate was also prepared, to provide a FAC of either 1 mM (human THIK-1) or 3 mM (mouse THIK-1, TREK-1, TWIK-2) thallium sulphate (in assay buffer). For Kv2.1, the FAC of thallium sulphate was 2.6 mM and this was prepared in assay buffer containing potassium sulphate (Sigma P9458; FAC 23.3 mM). Following 60 min of dye loading, 10 μl from the 3x FAC compound plate was added into the cell plate, which was then incubated for 15 min at room temperature in the dark. Using a fluorescent imaging plate reader (FLIPR) tetra plate reader, 15 μl thallium stimulus solution was added to the cell plate and the fluorescence signal recorded every second for 1 min (excitation: 470–495 nm, emission: 515–575 nm). Responses were normalised to baseline and the initial slopes of the thallium induced fluorescence responses (36–38 s; baseline corrected) were used to calculate the compound induced inhibition of constitutive (THIK-1, TREK-1, TWIK-2) or potassium sulphate induced activity (Kv2.1).

For the functional wash-off assay, plates were tested in parallel that were run as standard (no wash) with plates that had undergone an additional assay buffer wash and incubation period following compound addition. Briefly, after a 15 min compound incubation, plates were either moved to the FLIPR for immediate thallium addition (standard) or the plates were washed with assay buffer to remove compound solutions (50 μl/well addition and removal), 30 μl/well assay buffer added and the plates incubated for 60 min, followed by thallium addition on the FLIPR (60 min wash).

### 2.6 Q-patch automated electrophysiology assay

THIK-1 mediated currents were measured using the Q-Patch 48 automated electrophysiology platform (Sophion, Denmark). Experiments were performed at room temperature and in whole cell, single-whole configuration. The extracellular solution was composed of (in mM): NaCl 135, KCl 5, CaCl_2_ 2, HEPES 10, MgCl_2_ 2, d-glucose 10 (pH adjusted to 7.4 using NaOH). The intracellular solution was composed of (in mM): NaCl 5, KCl 135, CaCl_2_ 2, EGTA 11, MgATP 2, HEPES 10 (pH adjusted to 7.2 using KOH). The osmolality of both solutions was adjusted to 305–310 mOsm (Advanced Instruments 3320 micro-osmometer). Test compounds were prepared in DMSO (333x FAC) and then diluted in extracellular solution to provide the FAC (0.3% DMSO). TPA was prepared in extracellular solution (FAC 500 μM, 0.3% DMSO) and used as a reference inhibitor. After reaching whole cell configuration, cells were maintained at a holding potential of −60 mV, followed by a depolarising step to +40 mV for 500 ms to activate THIK-1. Each cell was exposed to 5x liquid additions (minimum of 15 sweeps/liquid addition) with a single compound concentration tested/cell: 1x EC solution (baseline), 2x compound, 2x TPA. Current amplitude was extracted at the end of the depolarising voltage step and data was rundown corrected using the average of the DMSO negative control cells. Concentration response curves were compiled using data from numerous cells within an experiment and compounds were tested in 2 independent experiments, resulting in 2–3 data points/concentration. Resulting potency values from individual experiments were all within 3-fold of the mean.

### 2.7 Competitive binding

From within the same chemotype as C101248, a structurally related compound (C101505) was tritiated for use in radioligand binding studies (specific activity 14 Ci/mmol). All radioligand binding assays were performed by Gifford Bioscience (Birmingham, UK). Cells were grown to approximately 90% confluence, washed twice with PBS and once with PBS with sucrose (10%) solution that was removed before the flasks were stored at −80 °C. Upon thawing, ice-cold lysis buffer (50 mM Tris-HCl; 5 mM MgCl_2_; 5 mM EDTA; protease inhibitor cocktail) was added to the flasks for 5 min (4 °C) and the cell layer scraped into collection tubes. The lysate was centrifuged at 13,000 g for 10 min (4 °C) to pellet the membranes. The membrane pellet was resuspended in wash buffer (50 mM Tris-HCl; 5 mM MgCl_2_; 5 mM EDTA) and recentrifuged. The pellet was then resuspended in wash buffer containing sucrose (10%) as a cryoprotectant, aliquoted and stored at −80 °C. A sample of the homogenate was analysed for protein content using a BCA protein assay kit. On the day of the assay, membrane pellets were thawed and resuspended in assay buffer (50 mM Tris, 5 mM MgCl_2_, 0.1 mM EDTA, pH 7.4). Compound solutions were prepared at 200x FAC in DMSO and then diluted with assay buffer to 5x FAC (2.5% DMSO). The binding assay was carried out in polypropylene 96 well plates in a final volume of 250 μl/well. To each well was added 150 μl of membranes and 50 μl of C101248 or buffer alone. The plate was incubated at room temperature for 30 min before the addition of 50 μl radioligand solution (at approximately the K_D_; diluted in assay buffer). The plate was then incubated at 30 °C for 120 min with agitation. The incubations were stopped by vacuum filtration onto pre-soaked (0.1% PEI in wash buffer) GF/C filters using a 96-well FilterMate harvester, followed by 6x washes with ice-cold wash buffer. Filters were then dried under a warm air stream, sealed in polyethylene, scintillation cocktail added, and the radioactivity counted in a MicroBeta TriLux counter (Wallac). Non-specific binding was subtracted from total binding for each compound concentration, to give specific binding. The in vitro binding affinity (K_i_) of C101248 was calculated from the experimental IC_50_ value using the Cheng-Prusoff equation ((Cheng and Prusoff, 1973) K_i_ = IC_50_/(1 + ([L]/K_D_)), where [L] was the radioligand concentration in the displacement assay and K_D_ was the radioligand dissociation constant determined from previous saturation binding studies.

### 2.8 Electrophysiology experiments

#### 2.8.1 Patch clamp of HEK-hTHIK-1 cells

Cells were plated onto uncoated, sterile glass coverslips at a density of 1 × 10^6^ cells/ml in a droplet of approximately 200 μl and incubated at 37 °C/5% CO_2_ before use at least 24 h later. Coverslips were transferred to the microscope stage chamber and cells visualised under IR-DIC illumination. The chamber was gravity perfused with a solution containing (in mM): 140 NaCl, 2.5 KCl, 10 HEPES, 1 NaH2PO4.H20, 2 CaCl_2_, 1 MgCl_2_, 10 glucose, with pH adjusted to 7.4 using NaOH and osmolarity adjusted to 320 mOsM using sucrose. The chamber was fed from a rapid perfusion exchanger, which continuously perfused square, multi-barrelled glass tubes, using laminar flow. Cells were patched using micropipettes filled with a solution containing (in mM): 125 KCl, 4 NaCl, 1 CaCl_2_, 10 HEPES, 10 EGTA, 4 MgATP, 2 Na_2_ATP, 2 MgCl_2_, 0.5 Na_2_GTP, pH adjusted to 7.1 with KOH and osmolarity adjusted to 320 mOsm with sucrose. The liquid junction potential was calculated to be 4.8 mV and was not corrected for. Cells were lifted from the coverslip and positioned directly in the perfusion flow approximately 100 μm from the glass perfusion tip. Voltage protocols were coupled to the solution exchanger from the trigger output. Cells were held at −50 mV and an 800 ms voltage ramp was applied from −120 to +50 mV every 1 s. Data were acquired at 5 kHz and filtered at 1 kHz. The solution was switched, using the rapid perfusion exchanger, between vehicle and 50 nM, 0.5 μM or 5 μM C101248 for 4 min. C101248 was prepared from a 10 mM stock in DMSO with ≤0.05% DMSO in the final solutions. The current at 50 mV from each sweep was measured and plotted against time. Curves were fitted in GraphPad Prism to a one phase decay [Y= IF(X < X0, Y0, Plateau+(Y0-Plateau)*exp(-K*(X-X0))), where x = time, y = Y0 until X = X0 then decays down to plateau with one phase, K = rate constant]. τ values were calculated to determine the activation time constant.

#### 2.8.2 Patch clamp of microglia in acute hippocampal slices

Acute hippocampal slices (300 μm thick), from 3 to 4 months old mice, were prepared using a specially adapted protective slicing solution (Ting et al., 2014) containing (mM) 93 choline chloride, 20 HEPES, 30 NaHCO_3_, 2.5 KCl, 0.5 CaCl_2_, 10 MgCl_2_, 1.2 NaH_2_PO_4_, 25 glucose, 1 kynurenic acid, 5 Na-ascorbate, 3 Na-pyruvate, pH adjusted to 7.4, bubbled with 95% O_2_/5% CO_2_ and cooled at <4 °C, and a recovery solution containing (mM) 92 NaCl, 20 HEPES, 30 NaHCO_3_, 2.5 KCl, 2 CaCl_2_, 1 MgCl_2_, 1.2 NaH_2_PO_4_, 25 glucose, 1 kynurenic acid, 5 Na-ascorbate, 3 Na-pyruvate, pH set to 7.4 and bubbled with 95% O_2_/5% CO_2_

Immediately after slicing, slices were placed for 13 min in warm (33–35 °C) slicing solution and then transferred to recovery solution at room temperature until experimental usage. Brain slicing does not activate microglia for at least 4 h, as judged by cell morphology, motility and IL-1β release (Gyoneva and Traynelis, 2013; Kurpius et al., 2007). Slices were perfused with bicarbonate-buffered solution, at 34–36 °C for all experiments, containing (mM) 124 NaCl, 2.5 KCl, 26 NaHCO_3_, 1 NaH_2_PO_4_, 2 CaCl_2_, 1 MgCl_2_, 10 glucose, bubbled with 95% O_2_/5% CO_2_. Microglia were whole-cell clamped with electrodes containing K-gluconate based solution, comprising (mM) 130 K-glu, 4 NaCl, 1 CaCl_2_, 10 HEPES, 10 EGTA, 4 MgATP, 0.5 Na_2_GTP, pH set to 7.2 with KOH and osmolarity adjusted to 285 ± 5 mOsmol/ml. GFP-encoded microglia were identified by epifluorescence using an upright Olympus BX51 microscope equipped with a 4-wavelength high-power LED excitation light source (Thorlabs) in combination with infrared differential interference contrast video microscopy using a high-sensitivity Hamamatsu ORCA-Flash LT CMOS camera. Cells were whole-cell patch-clamped at depths between 60 and 120 μm below the slice surface using borosilicate glass pipettes with a tip resistance of ∼4–5 MΩ resulting in series resistances of <20 MΩ. Electrode junction potentials were compensated. Voltage-clamp experiments were performed at a holding potential of 0 mV to set a large driving force for [K^+^] to leave the cells and to avoid the measurement of potential non-selective cation channels. Current-voltage (I–V) relationships were from responses to 200 msec voltage steps ranging from −150 mV to +60 mV from a holding potential of −30 mV. Patch-clamp recordings in voltage- and current clamp modes were performed using an Axopatch 200B amplifier (Molecular Devices). Currents were filtered at 1 kHz, digitized (10 kHz) and analysed off-line using pClamp10 software.

### 2.9 IL-1β measurement

IL-1β from culture supernatants was measured with DuoSet ELISA kit (R&D Systems) following the manufacturer instructions.

### 2.10 Human tissue experiments

Human tissue samples were obtained from the Miami Brain Endowment Bank (10.13039/100006686University of Miami), the General section of the Douglas-Bell Canada Brain Bank (Douglas Bell Hospital Research Centre, Montreal, Quebec, Canada), the NIH NeuroBioBank at the University of Miami, Maryland Brain Bank and Mount Sinai/JJ Peters VA Medical Center NBTR, Tissue4Research, London Neurodegenerative Diseases Brain bank at King's College London (LNDG receives funding from the UK Medical Research Council and as part of the Brains for Dementia Research programme, jointly funded by Alzheimer's Research UK and the Alzheimer's Society), The Netherlands Brain Bank (NBB, Netherlands Institute for Neuroscience, Amsterdam) and the South West Dementia Brain Bank (SWDBB). The SWDBB is part of the Brains for Dementia Research Programme, jointly funded by Alzheimer's Research UK and Alzheimer's Society and is supported by Bristol Research into Alzheimer's and Care of the Elderly (BRACE) and the Medical Research Council. We would like to thank the SWDBB and all other brain banks for providing brain tissue for this study. All human tissues used in this study were collected from donors for whom a full written consent was obtained for use of material and clinical information in accordance with the World Medical Associations Declaration of Helsinki for Medical Research. Data and material were managed in compliance with the UK Human Tissue Act. Clinical records confirmed that donors with AD (n = 18; 15 female and 3 male) were diagnosed during life. Control donors (n = 17; 9 female and 8 male) had not been diagnosed with a CNS-related disorder and died from a non-CNS related causes. The median age of the AD donors was 88 years (range 67–96) with a median post-mortem delay of 8 h (range 4.5–48). The median age for the control donors was 79 years (range 42–103) with a post-mortem delay of 14.2 (3.8–94) h.

#### 2.10.1 Nuclear Enriched Transcript Sort Sequencing (NETSseq)

Protocols for nuclei isolation from tissue samples are essentially as previously described (Xu et al., 2018), see also supplementary information.

After nuclei isolation, re-suspended nuclei were fixed with 1% formaldehyde for 8 min at room temperature, and then quenched with 0.125 M glycine for 5 min. Nuclei were pelleted at 1000×*g*, 4 min, 4 °C, and then washed once with homogenisation medium and once with Wash Buffer (PBS, 0.05% TritonX-100, 50 ng/mL bovine serum albumin, 1 mM DTT, 10 U/μl Superase-In RNase Inhibitor). Nuclei were blocked with Block Buffer (Wash buffer with an additional 50 ng/mL BSA) for 30 min, incubated with NeuN mouse primary antibody (1:250, Millipore, MAB377) and IRF8-PE fluorescently conjugated antibody (1:125, eBioscience, 12-9852-82) for 1 h, and then washed three times with Wash Buffer with spins in between washes as described above. Nuclei were then incubated in Alexa Fluor 594 anti-mouse secondary antibody (1:250, Jackson Immuno research, 715-585-151), for 30 min and washed three times with Wash Buffer. All incubations steps were performed at room temperature. Primary, secondary and conjugated antibodies were diluted in Block Buffer. Prior to flow cytometry, nuclei were co-stained with DAPI to 0.01 mg/ml final concentration. Nuclei were analysed and sorted using the BD FACSAria Fusion (BD Biosciences, San Jose, CA, USA) flow cytometer with 355 nm, 488 nm, 561 nm, and 640 nm lasers. All samples were first gated using forward and side scatter to exclude debris, followed by a single nuclei DAPI gate to exclude aggregates. Neuronal nuclei were excluded by gating low on SSC-A vs FSC-A, before sorting microglial nuclei within a NeuN negative and IRF8 positive gate. Analysis was performed using FACSDiva (BD) or FlowJo software (FlowJo, LLC). To reduce RNA degradation all nuclei samples were sorted at 4 °C and stored at −80 °C, before being processed for RNA extraction. RNA quality was assessed as previously described (Sun et al., 2021). Gene expression quantification was performed with htseq-count v 0.6.1p1 using a custom annotation file to be able to quantify reads in both exons and introns of RefSeq genes. Differential gene expression analyses to identify changes between AD patients (n = 18) and non-demented controls (n = 17) were performed using DESeq (Robles et al., 2012).

#### 2.10.2 Immunohistochemistry (IHC) and in situ hybridization (ISH)

The immunohistochemical detection of THIK-1 was performed using a polyclonal antibody to human THIK-1 (Origene, TA338706). Conventional automated immunohistochemical methods (Agilent Envision Flexplus) lacked the necessary sensitivity for reliable detection of this low expressing target. Application of a tyramide amplification step (Agilent CSAII) allowed the visualization of THIK-1 in 5 μm formalin-fixed paraffin embedded (FFPE) sections of a parietal cortex tissue. Briefly, following de-paraffinization, heat-induced epitope retrieval (low pH for 20 min at 97 °C) and rehydration using an Agilent PTLink slide incubator, sections were blocked, and endogenous peroxidases were quenched. Sections were incubated with anti-THIK-1 and detection of bound antibodies was completed using subsequent incubations with HRP-conjugated secondary antibodies, fluorescyl-tyramide, anti-FITC-HRP and chromogenic detection. Glial marker proteins, GFAP (AbCam, ab53554) and Iba1 (AbCam, ab15690) were detected using conventional, automated immunohistochemical methods (Envision Flexplus).

To visualize THIK-1 mRNA in human brain, duplex RNAScope (ACD Biosystems, Biotechne) in situ hybridization was completed with probes to human THIK-1 (536871-c1) and Iba1 (433131-c1). Taking advantage of superior RNA preservation, 10 μm frozen sections of parietal cortex from four donors (n = 2 non-demented control and AD) were used.

### 2.11 Statistical analysis

For thallium influx, radiolabelled binding, and Q-Patch assays, data are presented as mean values ± standard deviation (SD) and were analysed with a four-parameter logistic equation using GraphPad Prism. For thallium and Q-Patch assays, percent inhibition was calculated by normalising data to TPA controls; for the radioligand binding assay, data is expressed as percent inhibition of specific binding of the radiolabelled tool compound.

For electrophysiology experiment in brain slices, data are presented as standard error of the mean (S.E.M.) and statistical significance was calculated with GraphPad Prism using unpaired *t*-Test between control and treated groups.

For assays measuring IL-1β, data are presented as standard error of the mean (S.E.M.) and statistical significance was calculated with GraphPad Prism using One-way ANOVA followed by Dunnett's multiple comparison test.

For NETSseq analysis microglia samples were selected to represent distinct donors from three brain regions (parietal and entorhinal cortex, middle temporal gyrus). Differential gene expression analyses were carried out using R Bioconductor's DESeq2 package; an analysis design was modelled to consider variability coming from gender, brain region and level of genomic DNA contamination. Specifically, the model was designed as ∼ Gender + Kit + Pct_intergenic + Sample_region + Disease.

## 3 Results

### 3.1 THIK-1 is specifically expressed in human microglia and upregulated in brains from AD donors

**Fig. 1:**
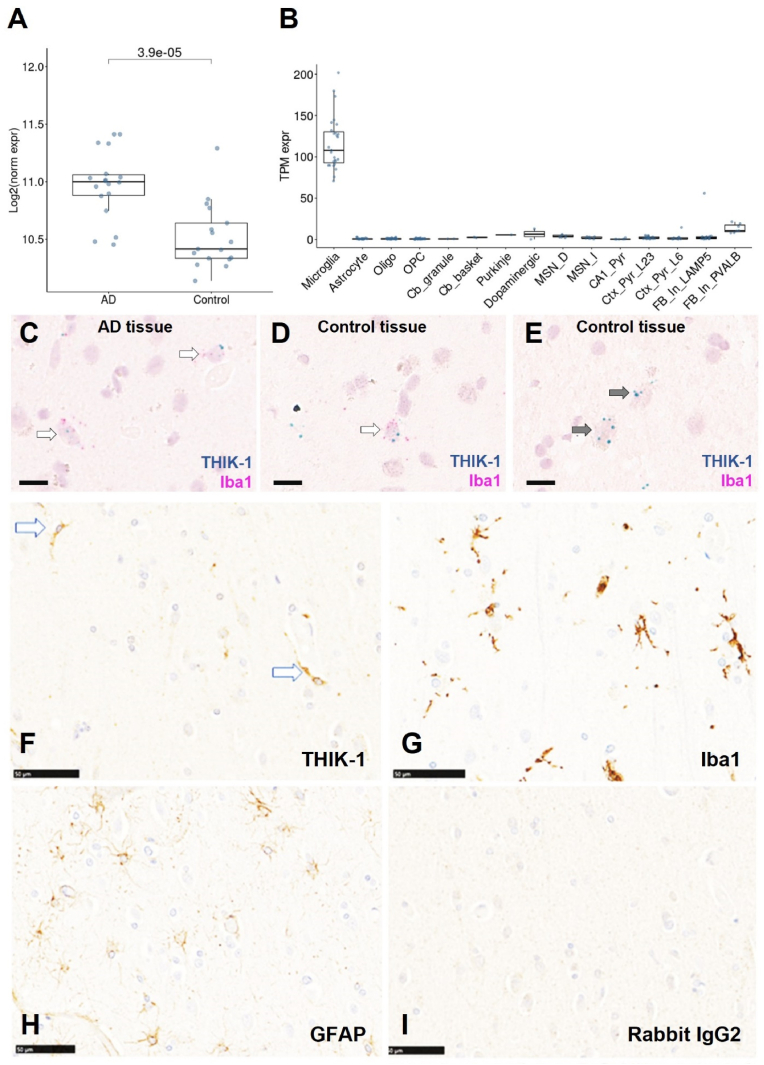
**THIK-1 mRNA and protein in human brain tissue.** Boxplots of elevated THIK-1 log_2_ normalised expression levels in AD group compared to control group (**A**). An unpaired *t*-test was used to calculate statistical significance between AD and control groups. Boxplots showing THIK-1 mRNA levels from control donors only in microglia, astrocytes, mature oligodendrocytes (Oligo), oligodendrocyte precursors cells (OPC), cerebellar granule cells (Cb_granule), cerebellar basket cells (Cb_basket), Purkinje cells, norepinephrine neurons from locus coeruleus (LC_NE), dopaminergic neurons from ventral tegmental area and substantia nigra (Dopaminergic), medium spiny neurons from direct and indirect pathway (MSN_D and MSN_I), CA1 hippocampal pyramidal neuron (CA1_Pyr), pyramidal neurons from layer 2 and 3 of various cortical regions (Ctx_Pyr_L23), pyramidal neurons from layer 6 of various cortical regions (Ctx_Pyr_L6), LAMP5 positive interneurons from various cortical regions, CA1, and dentate gyrus (FB_In_LAMP5), parvalbumin positive interneurons from various cortical regions (FB_In_PVALB) (**B**). Digital scans (40x Aperio Scanscope) showing using white arrows THIK-1 mRNA (blue hybridization signals) co-expressing with Iba1 mRNA in microglia (red) (**C-E**). Panels C and D show representative co-expression from two different donors, AD and control respectively. Panel E shows representative THIK-1 mRNA expression (blue signals) in Iba1 negative neurons, black arrows from control tissue. Scale bar, 10 μm. Digital scans (20x Hamamatsu Nanozoomer) showing representative THIK-1 and glial marker protein immunoreactivity (-ir) in FFPE sections of parietal cortex from a 90-years old female donor (non-demented control, Braak 3) (**F–I**). Panel F shows THIK-1-ir in glia with morphologies consistent with microglia. Panel G shows Iba-1-ir in microglia and H shows GFAP-ir in astrocytes in equivalent fields of view to that shown in F. Panel I shows very low levels of non-specific immunoreactivity resulting from incubation of sections with non-immune rabbit IgG under identical conditions. Scale bar, 50 μm.

Using NETSseq we compared the expression of THIK-1 in microglia-enriched nuclei isolated from the parietal and entorhinal cortex, and middle temporal gyrus of age matched non-neurodegenerative disease control donors (n = 17) versus donors affected by AD (n = 18). THIK-1 expression was significantly increased by 0.454 log_2_ fold change in nuclei isolated from AD compared to control brains (t (33) = 4.65, p < 3.9 × 10^−5^) (Fig. 1A). In addition, NETSseq showed that in control and AD brains, THIK-1 mRNA is highly enriched in microglia compared to several glia and neuronal cell types (Fig. 1B). ISH and IHC were used to neuroanatomically confirm localization of THIK-1. Fig. 1 C-E shows representative expression data from two different donors where THIK-1 mRNA co-expressed with Iba1 mRNA in microglia. A limited number of THIK-1 mRNA positive/Iba-1 mRNA negative neurons were also seen (Fig. 1E). For IHC, 5 μm FFPE sections were used with a protocol which included tyramide amplification. This protocol displayed marked immunoreactivity on HEK-hTHIK-1 cells with very low signal on parental cells (Supplementary Fig. 1). Fig. 1 F–I shows THIK-1 immunoreactivity alongside that of the astrocyte marker GFAP, and microglia marker Iba1, revealing greater morphological similarity between THIK-1 and Iba1 than between THIK-1 and GFAP.

### 3.2 Discovery of the THIK-1 inhibitor C101248

The HEK-hTHIK-1 thallium assay was miniaturised to a 1536 well plate format and a high-throughput screening campaign was performed using 666,120 compounds at UF Scripps Biomedical Research (USA). Using a cut-off of 31.7% inhibition (3x SD) gave 15,233 actives, a 2.29% hit rate from the primary screen. The activity of selected hits was confirmed in the HEK-hTHIK-1 assay, with off target or artefactual responses determined in parallel using the described CHO-hKv2.1 assay.

**Fig. 2:**
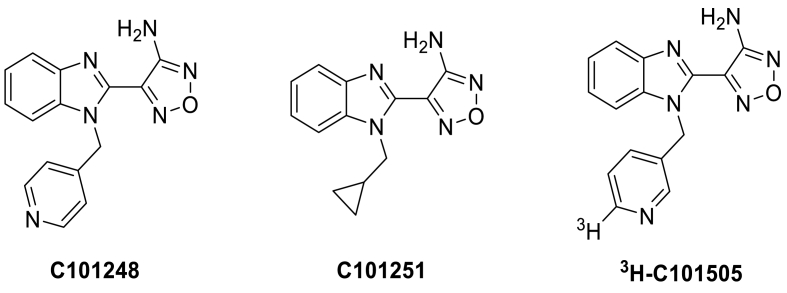
**Structures of THIK-1 inhibitors.** C101248, C101251 and the tritiated form of C101505 used in the competitive binding assay.

From the hit confirmation phase of this campaign, an active THIK-1 chemotype based on a 4-(benzo[*d*]imidazole-2-yl)-1,2,5-oxadiazol-3-amine motif was identified, as exemplified by compounds C101248 and C101251 (Fig. 2). The benzimidazole C101251 has previously been reported as a potent kinase inhibitor (Bandarage et al., 2009; Couty et al., 2013) and a co-crystal structure of C101251 in a PKA-S6K1 chimera complex has been documented [PDB: 4C36/(Couty et al., 2013)] confirming that C101251 binds in the ATP pocket of p70S6K1 with the 1,2,5-oxadiazol-3-amine element acting as a hinge binding motif. In the same publication, the benzyl analogue of C101251 was demonstrated to be inactive against p70S6K1(IC50 > 10 μM). In the protein crystal structure of C101251, it was shown that the cyclopropylmethyl group fits “snugly” into a hydrophobic pocket, defined by Gly50, Tyr54, Val57, and Phe327 in p70S6K1. It was postulated that this pocket is likely too small to accommodate the bulkier benzyl group, leading to loss of kinase activity. Based on this observation, we hypothesized that the 4-pyridylbenzyl unit of C101248 would also be too bulky for any kinase interaction. To confirm this, we profiled the pyridyl compound C101248 against seven kinases, including the kinases that were inhibited by the cyclopropyl compound C101251. As anticipated, no kinase inhibition was observed for C101248 (IC_50_ > 10 μM) for all kinases tested (Supplementary Table 1).

C101248 was further profiled in a promiscuity screening panel, using radiometric binding assays, against a selection of 18 diverse targets at 10 μM (Supplementary Table 2). Of these, only the hDAT receptor showed a response above 50% inhibition at 10 μM concentration.

C101248 concentrations in brain, CSF, and plasma were measured at multiple times (0.25, 0.5, 1 and 2 h post dose) following a single oral administration (30 mg/kg) in male mice (n = 3). The profile of C101248 in brain and CSF generally mirrored those in plasma with a maximal concentration (C_max_) being achieved at 1 h in all matrices. Resulting unbound partition coefficient (Kp_u,u_) and CSF:unbound plasma values ranged from 0.7 to 1.5 and 0.8–1.2, respectively demonstrating good distribution to the CNS. Brain concentrations of C101248 relative to plasma were also evaluated in rats as part of CNS PK studies (10 or 30 mg/kg, 1, 2 or 4 h post dose). The Kp_u,u_ values of 0.55–0.95 further confirmed the CNS distribution of C101248.

### 3.3 C101248 is a potent and selective inhibitor of human and mouse THIK-1

**Fig. 3:**
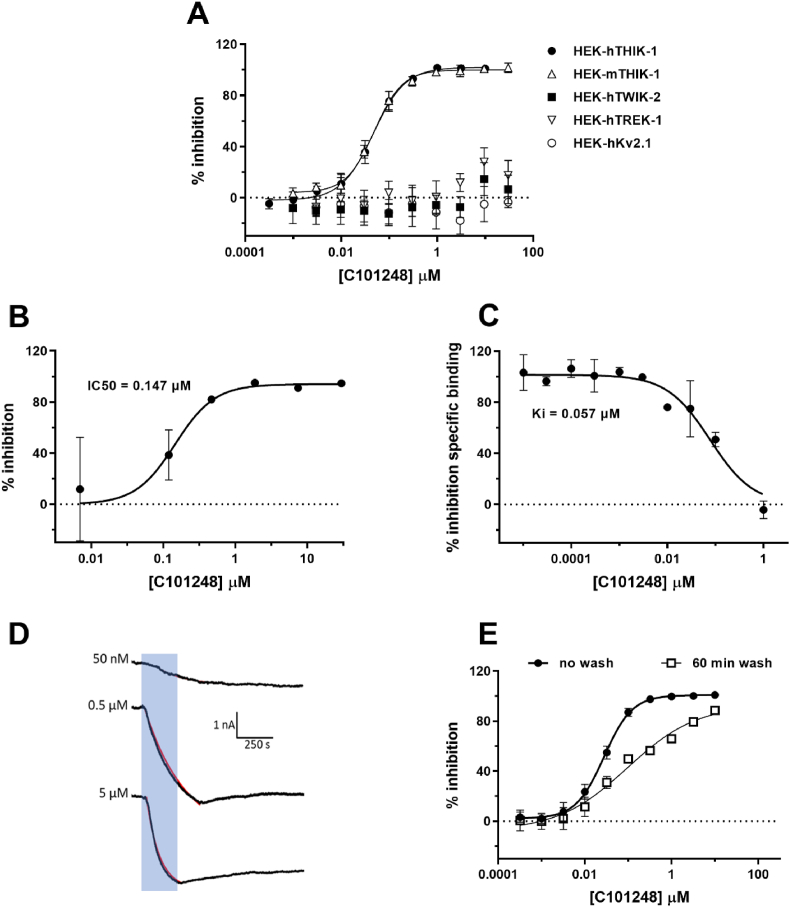
**Pharmacological characterization of C101248.** C101248 modulation of constitutive activity in HEK-hKCNK13, -mKCNK13, -hTWIK-2, -hTREK cells, and potassium sulphate-induced Kv2.1 activity was examined in a FLIPR thallium flux assay (**A**). The data shown is the averaged responses (±SD) normalised to TPA controls from 2 independent experiments, each performed in duplicate. Inhibition of THIK-1 mediated currents in HEK-hTHIK-1 by C101248 using the Qpatch 48 platform (**B**). Current amplitude was extracted at the end of the depolarising voltage step and the degree of inhibition normalised to TPA controls. Responses were averaged (±SD) from 2 independent experiments (providing 2–3 data points/concentration). C101248 displacement of radioligand binding to THIK-1 using membranes from HEK-hKCNK13 cells (**C**). Responses represents the average % inhibition of specific binding of the tool compound (C101505) (±SD) from a single experiment performed in duplicate. Current amplitude plotted over time showing the time course of C101248 inhibition (**D**). Examples of currents recorded at 50 mV before, during (shaded area) and after application of C101248, applied using a rapid perfusion exchanger for 4 min: 50 nM; n = 5, 0.5 μM; n = 3, 5 μM; n = 4. Red lines show a one phase decay fit for calculation of the activation time constant (τ). Comparison of C101248 potency to inhibit THIK-1 constitutive activity using the standard thallium flux protocol (no wash) or a 60-min wash protocol (60 min wash) (**E**). The data represent the averaged responses (±SD) normalised to TPA controls from 2 independent experiments, each performed in quadruplicate.

In the thallium influx assay, HEK-hTHIK-1 demonstrated clear constitutive activity that was fully inhibited by the non-selective potassium channel blocker TPA (IC_50_ = 4.7 μM) (Supplementary Fig. 2). C101248 produced a concentration dependent inhibition of THIK-1 constitutive activity, with an IC_50_ of 0.046 μM and maximum inhibition of 102% (Fig. 3A). C101248 demonstrated equipotent activity at mouse THIK-1, with an IC_50_ of 0.049 μM and 100% maximum inhibition. To assess the selectivity profile of C101248, activity was also examined at the two-pore domain potassium channels, TREK-1 and TWIK-2, as well as the voltage gated potassium channel Kv2.1. In HEK-hTWIK-2 and hTREK-1 cells, C101248 demonstrated minimal inhibition of constitutive activity, producing 7 and 18% inhibition, respectively (at 30 μM, compared to TPA controls). Similarly, in potassium sulphate stimulated CHO-Kv2.1 cells, C101248 produced no inhibition of thallium flux (−3% inhibition at 30 μM).

C101248 activity at human THIK-1 was also examined using the Q-Patch automated electrophysiology platform. In HEK-hTHIK-1 cells, C101248 fully inhibited basal currents with an IC_50_ value of 0.147 μM and maximum inhibition of 94% (Fig. 3B).

To assess the binding affinity of C101248 for THIK-1, the compound was examined in a competition binding assay with a structurally related radiolabelled compound (C101505, Fig. 3C). Full displacement of the radioligand was observed in this assay, with an IC_50_ of 0.077 μM. Using the Cheng-Prusoff equation (Cheng and Prusoff, 1973), the K_i_ for C101248 was determined to be 0.057 μM.

### 3.4 Kinetic characterisation of C101248

To assess the functional kinetics of C101248, we carried out whole-cell manual patch-clamp experiments on HEK-hTHIK-1 cells using a rapid perfusion exchanger. Activation time constants (τ) were calculated at 2 concentrations of C101248. Mean τ at 0.5 μM was 217.4 ± 61.4 s and was 83.4 ± 17.2 s at 5 μM (Fig. 3D). The τ for 0.05 μM C101248 could not be calculated as the onset of the inhibition was too slow to achieve an appropriate curve fit. As expected, τ decreased at the higher concentration indicating a faster on-rate of the compound. Off-rates could not be calculated due to limited recovery of current over a 20 min wash period. Overall, C101248 produced a rapid, concentration-dependent inhibition of THIK-1 current.

To further examine the dissociation of C101248 from THIK-1, the compound was tested in a modified thallium flux “wash-off” assay. In this assay, compounds were removed from the HEK-hTHIK-1 cell plate after the 15 min incubation and the cells were washed with buffer and incubated for 60 min (in buffer) prior to thallium addition (60 min wash). C101248 potency following the 60 min wash was compared to data from a standard thallium flux assay performed in parallel (15 min compound incubation, immediate thallium addition; no wash). Compared to the standard format, a 60 min wash-off produced a rightward shift in the concentration response curve for C101248 (Fig. 3E), with 3.4-fold lower potency observed (IC_50_ values: 0.028 μM (no wash) and 0.096 μM (60 min wash). The maximum inhibition achieved was similar under both conditions (101% (no wash) and 93% (60 min wash)). The C101248 inhibition curve from the 60 min wash had a Hill slope (n_H_) that was considerably shallower than in the no wash (n_H_ 0.54 compared to 1.3) and had a more biphasic profile.

### 3.5 C101248 inhibits THIK-1 K^+^ currents in microglia in situ

**Fig. 4:**
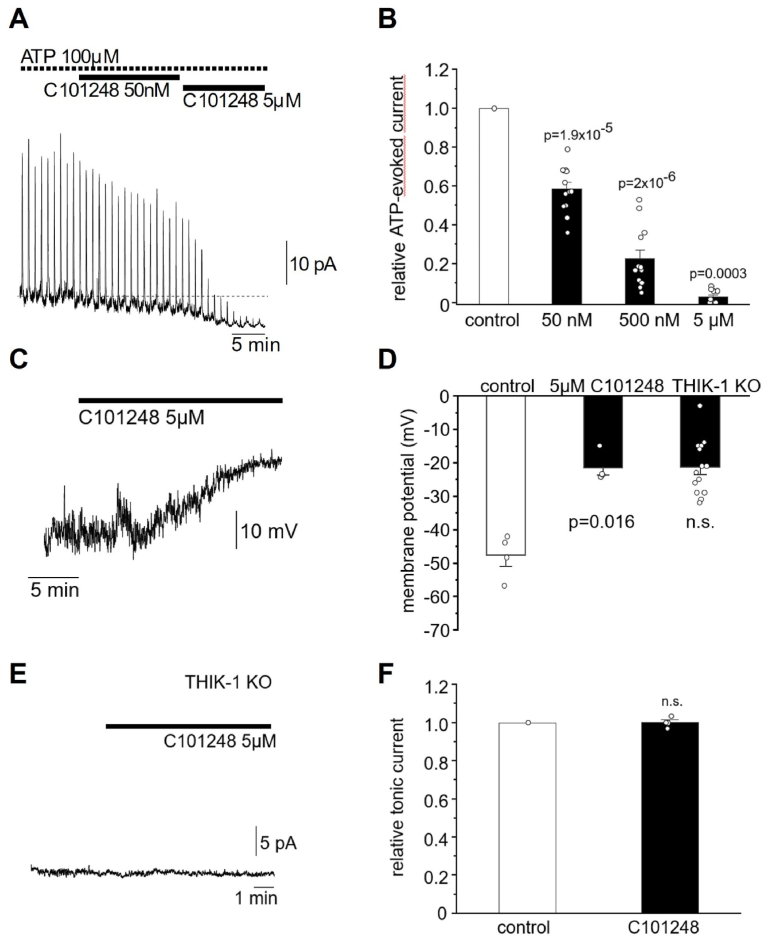
**Inhibition of microglial THIK-1 K**^**+**^**currents in acute hippocampal slices.** Specimen trace showing the dose-dependent inhibition by consecutive applications of 0.05 μM and 5 μM of C101248 on 100 μM ATP-evoked THIK-1 currents in microglia voltage-clamped at 0 mV (**A**). Note suppression of baseline current reflecting blockade of constitutive THIK-1 activity. Quantification of inhibitory potency by 0.05 μM, 0.5 μM and 5 μM C101248 on 100 μM ATP-evoked THIK-1 currents, normalised to internal controls of the same cell prior to blocker wash-in (**B**). Example recording in current-clamp mode illustrating the depolarization of microglial resting membrane potential by 5 μM C101248 (**C**). Quantification of resting membrane voltages with C101248 in wild-type mice in comparison to aged matched THIK-1 KO mice (**D**). Example showing the lack of effect on constitutively active baseline current on applying a saturating dose of 5 μM C101248 in microglia voltage-clamped at 0 mV from THIK-1 KO mice compared to normalised internal controls in the absence of blocker (**E and F**). Data are shown as means ± SEM.

To study the effect of C10248 in native brain tissue, THIK-1 currents were measured in microglia in acute hippocampal brain slices from adult wild-type mice by whole-cell patch clamp electrophysiology. Microglia generated robust P2Y_12_-activated THIK-1 K^+^ currents triggered by consecutive local puff-application of 100 μM ATP, which were dose-dependently inhibited by C101248 (0.05 μM: t(12) = 12.598 p = 2.8 × 10^−8^; 0.5 μM: t(11) = 16.78 p = 3.5 × 10^−9^; 5 μM: t(9) = −88.09 p = 1.59 × 10^−14^) (Fig. 4A and B). Experiments were performed by bath applying an initial sub-maximal concentration (0.05 μM or 0.5 μM) until reaching steady-state responses, followed by a saturating dose of 5 μM C101248. As seen from the specimen traces in Fig. 4A, C101248 blocked both the ATP-evoked THIK-1 currents and suppressed the constitutive THIK-1 activity, as indicated by the concomitant suppression in baseline current. No ATP-evoked residual current was elicited in the presence of a saturating dose of C101248, indicating activation of only THIK-1.

To investigate the effect of C101248 on constitutive THIK-1 K^+^ currents and the contribution to the microglial resting membrane potential, changes in membrane voltage were measured in current-clamp mode in the absence of externally applied ATP. At a saturating dose, C101248 (5 μM) significantly depolarised microglial resting membrane potential to the same level as measured in microglia from THIK-1 KO mice (t(3) = −4.92 p = 0.016) (Fig. 4C and D). Consistently, C101248 led to no further changes in baseline membrane current in THIK-1 KO mice (Fig. 4E and F), demonstrating a lack of effect on other constitutively active resting conductance in microglia. Likewise, C101248 had no effect on inward or outward currents induced by depolarising voltage steps ranging from −150 mV to +60 mV (Supplementary Fig. 3).

### 3.6 C101248 inhibits NLRP3-dependent release of IL-1β

Following the functional characterization of C101248 on THIK-1 conductivity, we tested the compound in various NLRP3-dependent inflammasome protocols.

**Fig. 5:**
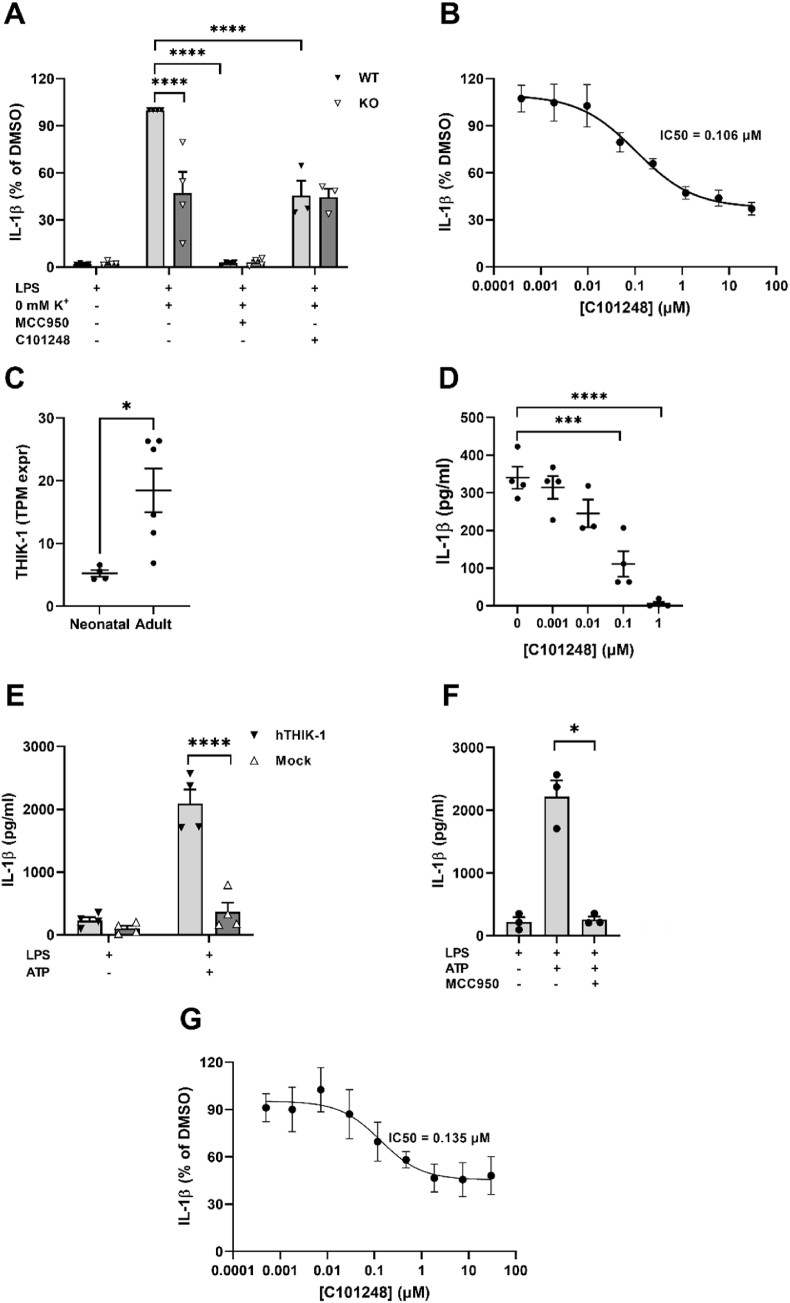
**THIK-1 inhibition prevents NLRP3-dependent release of IL-1β.** Activation of NLRP3 inflammasome in LPS-primed neonatal microglia cultures by exposure to K^+^-free buffer (**A** and **B**). THIK-1 depletion, MCC950 (1 μM), or C101248 (10 μM) prevented the release of IL-1β (**A**). Data are presented as % of DMSO-treated microglia isolated from wild-type mice and represent the mean ± S.E.M. of four (**A**) or six (**B**) independent experiments carried out in duplicates. Comparison of THIK-1 mRNA levels in neonatal vs adult cultured microglia (**C**). Data are presented as mean ± S.E.M. from at least four independent cultures. Statistical difference was calculated by unpaired *t*-test. Effect of C101248 on ATP-induced IL-1β release from microglia isolated from adult wild-type mice (**D**). Statistical differences were calculated using a one-way ANOVA, post hoc Dunnet's. Activation of NLRP3-dependent inflammasome in differentiated THP1 cells in the presence of MCC950 (1 μM), or C101248 (**E-G**). P < 0.05 = *; p < 0.001 = ***; p < 0.0001 = ****.

Initially we developed a protocol with neonatal primary mouse microglia in which LPS-primed cells were exposed to K^+^-free buffer to promote K^+^ efflux. This protocol produced an increase in IL-1β release that was significantly attenuated by addition of the NLRP3 inhibitor MCC950 (F(3,22) = 71.60, p < 0.0001) (Fig. 5A). Genetic deletion of THIK-1 reduced IL-1β release by 55% (F(1, 22) = 9.67, p = 0.0051) similar to the effect seen with 10 μM C101248 ((F(3,22) = 71.60, p < 0.0001) in wild type microglia. However, C101248 had no impact on K^+^-free buffer-induced release of IL-1β in THIK-1 KO microglia. In wild-type microglia C101248 produced a potent dose-dependent inhibition of IL-1β release following exposure to K^+^-free buffer (IC_50_ = 0.106 μM) (Fig. 5B).

When the NLRP3-activation protocol was switched to an LPS/ATP-induced release of IL-1β, blocking THIK-1 either genetically or pharmacologically had no effect on IL-1β levels (data not shown). We reasoned that this was due to the immature nature of microglia isolated from neonatal brains, leading us to develop a protocol with microglia isolated from adult mice. To note, adult microglia displayed a 3.5-fold increase in THIK-1 expression compared to neonatal microglia (t(8) = 3.08, p = 0.0163) (Fig. 5C). In the adult microglial system C101248 significantly attenuated LPS/ATP-induced IL-1β release at 0.1 and 1 μM (F(4,14) = 25.82, p <0.0002 (0.1 μM) and p < 0.0001 (1 μM)) (Fig. 5D).

To investigate the inhibition of THIK-1 in a human cell-based system we used the human monocytic cell line THP1. However, the activation of NLRP3 in THP1 cells with ATP produced a limited and variable release of IL-1β (Fig. 5E, mock-transfected cells). To overcome this limitation, which has been previously described (Redondo-Castro et al., 2018), we tested the effect of overexpressing human THIK-1. THIK-1 overexpression was functionally validated by an increase of thallium influx that was completely blocked by TPA (IC_50_ = 6.8 μM) (Supplementary Fig. 4). Interestingly, the overexpression of THIK-1 promoted a significant increase of IL-1β release compared to mock transfection (F(3,12) = 46.31, p < 0.0001) following ATP stimulation, which was completely blocked by MCC950 (F(1,2) = 47.96, p < 0.0001) (Fig. 5E and F). In this NLRP3-dependent inflammasome model, C101248 produced a potent concentration-dependent inhibition of IL-1β release (IC_50_ = 0.135 μM) (Fig. 5G).

## 4 Discussion

In this report we show how NETSseq has revealed the selective microglial expression of the K^+^ two pore domain channel (K2P) THIK-1 in human post-mortem cortex, while demonstrating increased expression of this channel in microglia isolated from donors affected by AD compared to non-demented control donors. Based on these findings, herein we disclose the discovery and characterisation of C101248, the first selective small-molecule inhibitor of THIK-1. We show that C101248 is a potent blocker of constitutive and ATP-evoked THIK-1 K^+^ currents with no detectable activity on closely related K2P channels or a selection of other K^+^ channels expressed in microglia. Using C101248 as a molecular probe, we determine, for the first time, that the activation of NLRP3-dependent inflammasome can be prevented by the selective pharmacological inhibition of THIK-1.

THIK-1 is an understudied member of the K2P family, and to the best of our knowledge, no selective blockers of this ion channel have been published to date. In this context, we screened 666,120 compounds in a FLIPR thallium influx assay and identified the benzimidazole C101248 as a potent and selective tool molecule to interrogate the in vitro pharmacology of THIK-1. Of note, C101248 was tested against several kinases and a promiscuity panel to de-risk potential liabilities and no concerning activity was highlighted.

Thallium influx and Q-patch assays showed that C101248 inhibited both human and mouse THIK-1 constitutive activity with a potency 100 times higher than that of tetrapenthylammonium chloride (TPA) which, despite its promiscuity, is commonly used tool to investigate the K2P family (Piechotta et al., 2011; Schewe et al., 2016). We confirmed the broad activity of TPA across K2P family members TREK-1 and TWIK-2 as well as on voltage-gated channels Kv2.1 (data not shown). This promiscuity is not surprising given the lipophilic, cationic nature of TPA but unfortunately renders it a tool of limited use. In contrast, even at 30 μM concentration, C101248 showed only a marginal inhibition of the closest THIK-1 homologues TREK-1 (18%) and TWIK-2 (7%) and had no effect on the Kv2.1 channel. Kinetic investigation of THIK-1 inhibition revealed that C101248 displayed a rapid association and a slow dissociation rate with an unchanged inhibition of THIK-1 current, as determined by manual patch clamp, following the removal of the compound (>20 min). Similarly, the inhibitory activity of the C101248 in the thallium influx assay was maintained for 60 min following wash-off (Fig. 3). This rapid inhibition of THIK-1 by C1012148 allows for a reduced preincubation time in in-vitro settings. On the other hand, the prolonged effect of C101248 suggests that the compound would continue to inhibit THIK-1 for at least an hour following its clearance from the CNS.

Electrophysiological recordings from microglia in acute hippocampal slices confirmed the ability of C101248 to block THIK-1, both tonic and ATP-evoked K^+^ currents (Fig. 4). Consistently, depletion of THIK-1 completely abrogates ATP-evoked K^+^ current in microglia (Madry et al., 2018b). Importantly, saturating concentrations of C101248 had no effect on voltage-dependent currents or on other tonic microglial current in THIK-1 KO mice. This supports the selectivity profile of our THIK-1 inhibitor against the most prominent ion channels that are constitutively active in microglial plasma membranes.

Reduction of intracellular K^+^ is a common event during NLRP3-dependent inflammasome activation following stimuli such as ATP, nigericin and silica crystals (Muñoz-Planillo et al., 2013; Pétrilli et al., 2007). Recently, Di and colleagues (Di et al., 2018) showed that TWIK-2, one of the most prominent K2P family member expressed in bone marrow-derived macrophages (BMDMs), is responsible, at least in part, for ATP-induced K^+^ efflux and NLRP3 inflammasome activation in macrophages. On the other hand, Madry and colleagues (Madry et al., 2018b) suggested a pivotal role of THIK-1 in ATP-evoked K^+^ efflux and consequently, activation of an inflammasome response in microglia. More recently, this was confirmed in a series of experiments carried out using microglia from dissociated cultures (Drinkall et al., 2022). These observations however are based on the use of either cellular systems isolated from constitutive THIK-1 KO mice or non-selective K^+^ channel blockers such as TPA and quinine. In this study we determined that the selective pharmacological inhibition of THIK-1 prevented NLRP3-dependent release of IL-1β in cultured microglia (Fig. 5). The removal of extracellular K^+^ in neonatal microglial cultures activated a NLRP3-dependent inflammasome that was partially prevented by the depletion of THIK-1 and inhibited to a similar magnitude by C101248. Furthermore, and most likely a result of the selectivity of our inhibitor, C101248 had no effect on microglia that lacked THIK-1 expression. When using microglial cultures isolated from adult mice, in which THIK-1 expression is 3.5-fold higher than neonatal microglia, ATP-induced IL-1β was almost completely prevented by pre-treatment with C101248. This is in line with data showing a marked reduction of ATP-induced IL-1β release from adult microglial cultures derived from THIK-1 KO mice (Drinkall et al., 2022).

To further support the contribution of THIK-1 to NLRP3 inflammasome activation in human immune cells, we showed that overexpressing this channel in THP-1 cells caused a marked increase of IL-1β release following ATP stimulation compared to mock transfected cells, which was completely prevented by the NLRP3 inhibitor MCC950 (Fig. 5E–G). In this system, C101248 concentration-dependently blocked the release of IL-1β with a potency comparable to equivalent inflammasome assays in mouse microglia. The observation that THP-1 cells required overexpression of THIK-1 to produce a robust release of IL-1β following ATP stimulation suggests that NLRP3 activation in human macrophages is less dependent on THIK-1 compared to microglia. This hypothesis is also supported by the observation that microglia isolated from human brains express 12.0- and 2.2-fold higher levels of THIK-1 compared to human monocytes and macrophages, respectively (Galatro et al., 2017). Interestingly, the depletion of THIK-1 also appears to prevent ATP-induced IL-1β release from microglia more profoundly than in BMDMs isolated from adult mice (Drinkall et al., 2022). In addition to these cell type differences, THIK-1 is expressed 5.8-fold higher in monocytes isolated from mice compared to cells isolated from humans (Gosselin et al., 2017) suggesting potential species differences between human and rodent.

NETSseq allowed us to highlight the microglial specific expression of THIK-1 in the human brain against many glia and neuronal cell types. This was confirmed at both the mRNA and protein level by ISH and IHC of human post-mortem parietal cortex tissue. Importantly, NETSseq also revealed, for the first time, that THIK-1 expression is increased in microglial nuclei isolated from donors affected by AD compared to non-disease controls, supporting the role of this channel in disease. This hypothesis is also supported by the recent identification of a SNP in THIK-1 as a potential risk factor in subpopulations of AD patients (Zhang et al., 2019). This is consistent with evidence showing the presence of NLRP3-dependent inflammasome activation in AD brains and the deleterious effect of this pro-inflammatory cascade in animal models of AD (Heneka et al., 2013; Ising et al., 2019).

In summary, here we report the identification and characterisation of the novel THIK-1 K^+^ channel inhibitor C101248. Based on the demonstrated function of THIK-1 in modulating NLRP3 inflammasome activation, together with its disease related changes in expression, we believe that molecules such as C101248 may have therapeutic utility in the treatment of neurodegenerative diseases where microglial mediated neuroinflammation is a key underlying driver of progression. In addition, the fact that THIK-1 is specifically expressed in microglia potentially favours the safety profile of a potent and selective THIK-1 inhibitor.

## CRediT authorship contribution statement

**Bernardino Ossola:** Conceptualization, Methodology, Investigation, Data curation, Writing – original draft. **Ali Rifat:** Methodology, Investigation, Data curation, Writing – original draft. **Anna Rowland:** Methodology, Investigation. **Helen Hunter:** Methodology, Investigation. **Samuel Drinkall:** Methodology, Investigation, Data curation. **Clare Bender:** Methodology, Investigation. **Mayida Hamlischer:** Methodology, Investigation. **Martin Teall:** Investigation. **Russell Burley:** Investigation. **Daneil F. Barker:** Methodology, Investigation. **David Cadwalladr:** Investigation. **Louise Dickson:** Conceptualization, Data curation, Writing – original draft. **Jason M.K. Lawrence:** Investigation. **Jenna R.M. Harvey:** Investigation, Data curation, Writing – original draft. **Marina Lizio:** Software, Investigation, Data curation, Writing – original draft. **Xiao Xu:** Methodology, Software, Investigation. **Edel Kavanagh:** Conceptualization. **Toni Cheung:** Methodology, Investigation, Writing – original draft. **Steve Sheardown:** Methodology, Investigation. **Catherine B. Lawrence:** Writing – review & editing. **Michael Harte:** Writing – review & editing. **David Brough:** Investigation, Writing – review & editing. **Christian Madry:** Conceptualization, Data curation, Writing – original draft. **Kim Matthews:** Writing – review & editing. **Kevin Doyle:** Investigation, Data curation, Writing – original draft. **Keith Page:** Methodology, Investigation, Data curation, Writing – original draft. **Justin Powell:** Methodology, Software. **Nicola L. Brice:** Conceptualization, Writing – review & editing. **Roland W. Bürli:** Conceptualization, Writing – original draft, Writing – review & editing. **Mark B. Carlton:** Conceptualization, Writing – review & editing. **Lee A. Dawson:** Conceptualization, Writing – original draft, Writing – review & editing.

## Declaration of competing interest

All authors except for Ali R, SD, CB, Michael H, DB, CM were employers of Cerevance Ltd at the time their contribution to this work. Ali R and CM were supported by Cerevance Ltd for this work. CB, Michael H, and DB have no interest to declare.

## Data Availability

Data will be made available on request.

## References

[bib1] Bandarage U., Hare B., Parsons J., Pham L., Marhefka C., Bemis G., Tang Q., Moody C.S., Rodems S., Shah S., Adams C., Bravo J., Charonnet E., Savic V., Come J.H., Green J. (2009). 4-(Benzimidazol-2-yl)-1,2,5-oxadiazol-3-ylamine derivatives: potent and selective p70S6 kinase inhibitors. Bioorg. Med. Chem. Lett.

[bib2] Barclay W., Shinohara M.L. (2017). Inflammasome activation in multiple sclerosis and experimental autoimmune encephalomyelitis (EAE). Brain Pathol..

[bib3] Carrillo-Jimenez A., Puigdellívol M., Vilalta A., Venero J.L., Brown G.C., StGeorge-Hyslop P., Burguillos M.A. (2018). Effective knockdown of gene expression in primary microglia with siRNA and magnetic nanoparticles without cell death or inflammation. Front. Cell. Neurosci..

[bib4] Cheng Y.-C., Prusoff W.H. (1973). Relationship between the inhibition constant (KI) and the concentration of inhibitor which causes 50 per cent inhibition (I50) of an enzymatic reaction. Biochem. Pharmacol..

[bib5] Colonna M., Butovsky O. (2017). Microglia function in the central nervous system during health and neurodegeneration. Annu. Rev. Immunol..

[bib6] Couty S., Westwood I.M., Kalusa A., Cano C., Travers J., Boxall K., Chow C.L., Burns S., Schmitt J., Pickard L., Barillari C., Mcandrew P.C., Clarke P.A., Linardopoulos S., Griffin R.J., Aherne G.W., Raynaud F.I., Workman P., Jones K., van Montfort R.L.M. (2013). The discovery of potent ribosomal S6 kinase inhibitors by high-throughput screening and structure-guided drug design. Oncotarget.

[bib7] Di A., Xiong S., Ye Z., Malireddi R.K.S., Kometani S., Zhong M., Mittal M., Hong Z., Kanneganti T.D., Rehman J., Malik A.B. (2018). The TWIK2 potassium efflux channel in macrophages mediates NLRP3 inflammasome-induced inflammation. Immunity.

[bib8] Drinkall S., Lawrence C.B., Ossola B., Russell S., Bender C., Brice N.B., Dawson L.A., Harte M., Brough D. (2022). The two-pore potassium channel THIK‐1 regulates NLRP3 inflammasome activation. Glia.

[bib9] Galatro T.F., Holtman I.R., Lerario A.M., Vainchtein I.D., Brouwer N., Sola P.R., Veras M.M., Pereira T.F., Leite R.E.P., Möller T., Wes P.D., Sogayar M.C., Laman J.D., den Dunnen W., Pasqualucci C.A., Oba-Shinjo S.M., Boddeke E.W.G.M., Marie S.K.N., Eggen B.J.L. (2017). Transcriptomic analysis of purified human cortical microglia reveals age-associated changes. Nat. Neurosci..

[bib10] Gosselin D., Skola D., Coufal N.G., Holtman I.R., Schlachetzki J.C.M., Sajti E., Jaeger B.N., O'Connor C., Fitzpatrick C., Pasillas M.P., Pena M., Adair A., Gonda D.D., Levy M.L., Ransohoff R.M., Gage F.H., Glass C.K. (2017). An environment-dependent transcriptional network specifies human microglia identity. Science.

[bib11] Gyoneva S., Traynelis S.F. (2013). Norepinephrine modulates the motility of resting and activated microglia via different adrenergic receptors. J. Biol. Chem..

[bib12] Heneka M.T., Kummer M.P., Latz E. (2014). Innate immune activation in neurodegenerative disease. Nat. Rev. Immunol..

[bib13] Heneka M.T., Kummer M.P., Stutz A., Delekate A., Schwartz S., Vieira-Saecker A., Griep A., Axt D., Remus A., Tzeng T.C., Gelpi E., Halle A., Korte M., Latz E., Golenbock D.T. (2013). NLRP3 is activated in Alzheimer's disease and contributes to pathology in APP/PS1 mice. Nature.

[bib14] Heneka M.T., McManus R.M., Latz E. (2018). Inflammasome signalling in brain function and neurodegenerative disease. Nat. Rev. Neurosci..

[bib15] Ising C., Venegas C., Zhang S., Scheiblich H., Schmidt S.v., Vieira-Saecker A., Schwartz S., Albasset S., McManus R.M., Tejera D., Griep A., Santarelli F., Brosseron F., Opitz S., Stunden J., Merten M., Kayed R., Golenbock D.T., Blum D., Latz E., Buée L., Heneka M.T. (2019). NLRP3 inflammasome activation drives tau pathology. Nature.

[bib16] Izquierdo P., Shiina H., Hirunpattarasilp C., Gillis G., Attwell D. (2021). Synapse development is regulated by microglial THIK-1 K ^+^ channels. Proc. Natl. Acad. Sci. USA.

[bib17] Jay T.R., von Saucken V.E., Landreth G.E. (2017). TREM2 in neurodegenerative diseases. Mol. Neurodegener..

[bib18] Jayaraj R.L., Azimullah S., Beiram R., Jalal F.Y., Rosenberg G.A. (2019). Neuroinflammation: friend and foe for ischemic stroke. J. Neuroinflammation.

[bib19] Jung S., Aliberti J., Graemmel P., Sunshine M.J., Kreutzberg G.W., Sher A., Littman D.R. (2000). Analysis of fractalkine receptor CX 3 CR1 function by targeted deletion and green fluorescent protein reporter gene insertion. Mol. Cell Biol..

[bib20] Kang D., Hogan J.O., Kim D. (2014). THIK-1 (K2P13.1) is a small-conductance background K+ channel in rat trigeminal ganglion neurons. Pflueg. Arch. Eur. J. Physiol..

[bib21] Kelley N., Jeltema D., Duan Y., He Y. (2019). The NLRP3 inflammasome: an overview of mechanisms of activation and regulation. Int. J. Mol. Sci..

[bib22] Kurpius D., Nolley E.P., Dailey M.E. (2007). Purines induce directed migration and rapid homing of microglia to injured pyramidal neurons in developing hippocampus. Glia.

[bib23] Kyrargyri V., Madry C., Rifat A., Arancibia-Carcamo I.L., Jones S.P., Chan V.T.T., Xu Y., Robaye B., Attwell D. (2020). P2Y13 receptors regulate microglial morphology, surveillance, and resting levels of interleukin 1β release. Glia.

[bib24] Li Q., Barres B.A. (2018). Microglia and macrophages in brain homeostasis and disease. Nat. Rev. Immunol..

[bib25] Li X.X., Zhang F. (2021). Targeting TREM2 for Parkinson's disease: where to go?. Front. Immunol..

[bib26] Li Y., Laws S.M., Miles L.A., Wiley J.S., Huang X., Masters C.L., Gu B.J. (2021). Genomics of Alzheimer's disease implicates the innate and adaptive immune systems. Cell. Mol. Life Sci..

[bib27] Madry C., Arancibia-Cárcamo I.L., Kyrargyri V., Chan V.T.T., Hamilton N.B., Attwell D. (2018). Effects of the ecto-ATPase apyrase on microglial ramification and surveillance reflect cell depolarization, not ATP depletion. Proc. Natl. Acad. Sci. U. S. A..

[bib28] Madry C., Kyrargyri V., Arancibia-Cárcamo I.L., Jolivet R., Kohsaka S., Bryan R.M., Attwell D. (2018). Microglial ramification, surveillance, and interleukin-1β release are regulated by the two-pore domain K+ channel THIK-1. Neuron.

[bib29] Muñoz-Planillo R., Kuffa P., Martínez-Colón G., Smith B.L., Rajendiran T.M., Núñez G. (2013). K+ efflux is the common trigger of NLRP3 inflammasome activation by bacterial toxins and particulate matter. Immunity.

[bib30] Pétrilli V., Papin S., Dostert C., Mayor A., Martinon F., Tschopp J. (2007). Activation of the NALP3 inflammasome is triggered by low intracellular potassium concentration. Cell Death Differ..

[bib31] Piechotta P.L., Rapedius M., Stansfeld P.J., Bollepalli M.K., Erhlich G., Andres-Enguix I., Fritzenschaft H., Decher N., Sansom M.S.P., Tucker S.J., Baukrowitz T. (2011). The pore structure and gating mechanism of K2P channels. EMBO J..

[bib32] Qiao Y., Wang P., Qi J., Zhang L., Gao C. (2012). TLR-induced NF-κB activation regulates NLRP3 expression in murine macrophages. FEBS (Fed. Eur. Biochem. Soc.) Lett..

[bib33] Rajan S., Wischmeyer E., Karschin C., Preisig-Müller R., Grzeschik K.H., Daut J., Karschin A., Derst C. (2001). THIK-1 and THIK-2, a novel subfamily of tandem pore domain K+ channels. J. Biol. Chem..

[bib34] Ransohoff R.M. (2016). How neuroinflammation contributes to neurodegeneration. Science.

[bib35] Redondo-Castro E., Faust D., Fox S., Baldwin A.G., Osborne S., Haley M.J., Karran E., Nuthall H., Atkinson P.J., Dawson L.A., Routledge C., Allan S.M., Freeman S., Brownlees J., Brough D. (2018). Development of a characterised tool kit for the interrogation of NLRP3 inflammasome-dependent responses. Sci. Rep..

[bib36] Robles J.A., Qureshi S.E., Stephen S.J., Wilson S.R., Burden C.J., Taylor J.M. (2012). Efficient experimental design and analysis strategies for the detection of differential expression using RNA-Sequencing. BMC Genom..

[bib37] Schewe M., Nematian-Ardestani E., Sun H., Musinszki M., Cordeiro S., Bucci G., de Groot B.L., Tucker S.J., Rapedius M., Baukrowitz T. (2016). A non-canonical voltage-sensing mechanism controls gating in K2P K+ channels. Cell.

[bib38] Simon D.W., McGeachy M.J., Bayir H., Clark R.S.B.B., Loane D.J., Kochanek P.M., Baylr H., Clark R.S.B.B., Loane D.J., Kochanek P.M. (2017). Neuroinflammation in the evolution of secondary injury, repair, and chronic neurodegeneration after traumatic brain injury. Nat. Rev. Neurol..

[bib39] Sun H., Monenschein H., Schiffer H.H., Reichard H.A., Kikuchi S., Hopkins M., Macklin T.K., Hitchcock S., Adams M., Green J., Brown J., Murphy S.T., Kaushal N., Collia D.R., Moore S., Ray W.J., English N.M., Carlton M.B.L., Brice N.L. (2021). First-time disclosure of CVN424, a potent and selective GPR6 inverse agonist for the treatment of Parkinson's disease: discovery, pharmacological validation, and identification of a clinical candidate. J. Med. Chem..

[bib40] Thonhoff J.R., Simpson E.P., Appel S.H. (2018). Neuroinflammatory mechanisms in amyotrophic lateral sclerosis pathogenesis. Curr. Opin. Neurol..

[bib41] Ting J.T., Daigle T.L., Chen Q., Feng G. (2014). Acute brain slice methods for adult and aging animals: application of targeted patch clamp analysis and optogenetics. Methods Mol. Biol..

[bib42] Toma C., Higa N., Koizumi Y., Nakasone N., Ogura Y., McCoy A.J., Franchi L., Uematsu S., Sagara J., Taniguchi S., Tsutsui H., Akira S., Tschopp J., Núñez G., Suzuki T. (2010). Pathogenic Vibrio activate NLRP3 inflammasome via cytotoxins and TLR/Nucleotide-Binding oligomerization domain-mediated NF-κB signaling. J. Immunol..

[bib43] Xu X., Stoyanova E.I., Lemiesz A.E., Xing J., Mash D.C., Heintz N. (2018). Species and cell-type properties of classically defined human and rodent neurons and glia. Elife.

[bib44] Zhang X., Zhu C., Beecham G., Vardarajan B.N., Ma Y., Lancour D., Farrell J.J., Chung J., Bellair M., Dinh H., Doddapeneni H., Dugan-Perez S., English A., Gibbs R.A., Han Y., Hu J., Jayaseelan J., Kalra D., Khan Z., Korchina V., Lee Sandra, Liu Y., Liu Xiuping, Muzny D., Nasser W., Salerno W., Santibanez J., Skinner E., White S., Worley K., Zhu Y., Beiser A., Chen Y., Cupples L.A., DeStefano A., Dupuis J., Farrell J., Farrer L., Lin H., Liu C.T., Lunetta K., Patel D., Sarnowski C., Satizabal C., Seshadri S., Sun F.J., Choi S.H., Banks E., Gabriel S., Gupta N., Bush W., Butkiewicz M., Haines J., Smieszek S., Song Y., Barral S., de Jager P.L., Mayeux R., Reitz C., Reyes D., Tosto G., Vardarajan B., Amad S., Amin N., Ikram M.A., van der Lee Sven, van Duijn C., Vanderspek A., Schmidt H., Schmidt R., Goate A., Kapoor M., Marcora E., Renton A., Faber K., Foroud T., Feolo M., Stine A., Launer L.J., Bennett D.A., Xia L.C., Hamilton-Nelson K., Jaworski J., Kunkle B., Martin E., Pericak-Vance M., Rajabli F., Schmidt M., Mosley T.H., Cantwell L., Childress M., Chou Y.F., Cweibel R., Gangadharan P., Kuzma A., Leung Y.Y., Lin H.J., Malamon J., Mlynarski E., Naj A., Qu L., Schellenberg G., Valladares O., Wang L.S., Wang W., Zhang N., Below J.E., Boerwinkle E., Bressler J., Fornage M., Jian X., Liu Xiaoming, Bis J.C., Blue E., Brown L., Day T., Dorschner M., Horimoto A.R., Nafikov R., Nato A.Q., Navas P., Nguyen H., Psaty B., Rice K., Saad M., Sohi H., Thornton T., Tsuang D., Wang B., Wijsman E., Witten D., Antonacci-Fulton L., Appelbaum E., Cruchaga C., Fulton R.S., Koboldt D.C., Larson D.E., Waligorski J., Wilson R.K., Schellenberg G.D., Pericak-Vance M.A., Lunetta K.L., Farrer L.A. (2019). A rare missense variant of CASP7 is associated with familial late-onset Alzheimer's disease. Alzheimer's Dementia.

