# Supplementary material for: Characterisation of C101248: A novel selective THIK-1 channel inhibitor for the modulation of microglial NLRP3-inflammasome
Source: Neuropharmacology. 2023 Feb 15;224:109330. doi: 10.1016/j.neuropharm.2022.109330 (PMC9841576; doi:10.1016/j.neuropharm.2022.109330)
Supplement: Multimedia component 1 [file mmc1.docx]

**Supplementary information for**

**Characterisation of C101248: a novel selective THIK-1 channel inhibitor for the modulation of microglial NLRP3-inflammosome**

Bernardino Ossola, Ali Rifat, Anna Rowland, Helen Hunter, Samuel Drinkall, Clare Bender, Mayida Hamlischer, Martin Teall, Russell Burley, Dan Barker, David Cadwalladr, Louise Dickson, Jason Lawrence, Jenna Harvey, Marina Lizio, Xiao Xu, Edel Kavanagh, Toni Cheung, Steve Sheardown, Catherine B. Lawrence, Michael Harte, David Brough, Christian Madry, Kim Matthews, Kevin Doyle, Keith Page, Justin Powell, Nicola L. Brice, Roland W. Bürli, Mark B. Carlton, Lee A. Dawson

Bernardino Ossola, dino.ossola@cerevance.com

**Supplementary Methods**

**Synthesis of C101248 and C101505**

***General Methods***. Nuclear magnetic resonance (NMR) spectra were recorded at 400 MHz at 298.2K. The chemical shifts (δ) are reported in parts per million. Spectra were recorded using a Bruker^®^ 400 AVANCE Instrument fitted with a 5mm iprobe or smart probe with instrument controlled by Bruker TopSpin 4.0.9 software. Mass spectra were recorded with a Shimadzu single quadrupole mass spectrometer using DUIS ionisation (flow rate 1.0 ml/min). Two mobile phases were used: mobile phase A: 0.02% aq. NH_3_, mobile phase B: CH_3_CN (gradient: 90-20% A in 6 min. High-resolution mass spectra were recorded on a Thermo Q Exactive Orbitrap MS with an ESI ion source. Data was recorded in both positive and negative mode applying the following instrument parameters: Spray Voltage: 3500 V; Aux gas heater temperature: 400 °C; capillary temperature: 300 °C; sheath gas flow rate: 53; Aux gas flow rate: 14; sweep gas flow rate: 3. mass range: 100-1500. Data was acquired using X-Calibur software. Purity was assessed by UPLC with UV detection (220 – 254 nm) using Shimadzu^®^ Nexera X2 UPLC controlled by Lab Solution software equipped with a XBridge Shield RP18 (2.1 × 50 mm, 5 μm) column operated at 50°C.  Mobile phase consisted of CH_3_CN mixed with H_2_O containing 0.037% CF_3_COOH. Melting Point was recorded by Shanghai Shengguang WRS-2A Melting Point Apparatus.

***Synthesis***. C101248 and C101505 were synthesized in two steps. The first step involved coupling of the oxadiazole derivative **2** to phenylene diamine **1** and subsequent ring closure while the resulting benzimidazole (**3**) was alkylated in a second step to form the desired products.

*4-(1H-benzo[d]imidazol-2-yl)-1,2,5-oxadiazol-3-amine* (**3**). A mixture of benzene-1,2-diamine (10.0 g, 92.5 mmol) and (3Z)-4-amino-N-hydroxy-1,2,5-oxadiazole-3-carboximidoyl chloride hydrochloride **2** (18.4 g, 92.5 mmol) in EtOH (300 ml) was stirred at 90°C for 12 h and cooled to RT. The resulting precipitate was collected by filtration to give 4-(1H-benzimidazol-2-yl)-1,2,5-oxadiazol-3-amine **3** (11 g, 59%,) as an off-white. ^1^H NMR (400 MHz, DMSO-d_6_): δ 13.69 (br. s, 1H), 7.98-7.50 (m, 2H), 7.33 (dd, J = 7.2, 17.3 Hz, 2H), 6.83 (s, 2H). MS ESI+: 202.1 [M + H]^+^. Analyt. UPLC: 99.4%.

*4-[1-(Pyridin-4-ylmethyl)benzimidazol-2-yl]-1,2,5-oxadiazol-3-amine* (C101248). A solution of 4-(1H-benzimidazol-2-yl)-1,2,5-oxadiazol-3-amine **3** (2.00 g, 9.94 mmol) in DMF (15 ml) was treated with 4-(chloromethyl)pyridine hydrochloride **4** (1.63 g, 9.94 mmol), Cs_2_CO_3_ (9.72 g, 29.82 mmol) and KI (1.65 g, 9.94 mmol), stirred at 120°C for 8 h and cooled to RT. The mixture was treated with H_2_O (30 ml) and the resulting precipitate was collected by filtration. The filter cake was triturated with several solvents in the following order 25 °C: EtOH (10 ml) for 1 h, EtOAc (10 ml) for 1 h, MeOH (10 ml) for 8 h and H_2_O (30 ml) for 8 h. The product was dissolved in CHCl_3_ (50 ml) and washed with a sat. aq. LiCl solution (250 ml). The organic layer was dried (Na_2_SO_4_) and filtered. The filtrate was evaporated to dryness to give 4-[1-(pyridin-4-ylmethyl)benzimidazol-2-yl]-1,2,5-oxadiazol-3-amine C101248 (930 mg, 81%) as a yellow solid. ^1^H NMR (400 MHz, DMSO) δ 8.48 (br. d, *J* ≈ 5.9 Hz, 2H), 7.90 (dd, *J* = 7.0, 2.2 Hz, 1H), 7.71 (br. dd, *J* = 6.6, 1.6 Hz, 1H), 7.38-7.45 (m, 2H), 7.08 (d, J = 5.9 Hz, 2H), 7.01 (s, 2H), 6.01 (s, 2H). MS ESI^+^: 293.3 [M + H]^+^. HR-MS (ESI) for C_15_H_13_N_6_O: 293.1145 (calc.), 293.1148 (found). Analyt. UPLC: 96.0%. Melting point: 240.0-240.5 °C.

*4-[1-(Pyridin-3-ylmethyl)benzimidazol-2-yl]-1,2,5-oxadiazol-3-amine* (C101505).

A stirred solution of 4-(benzimidazol-2-yl)-1,2,5-oxadiazol-3-amine **3** (0.10 g, 0.50 mmol) in DMF (5 ml) was treated with K_2_CO_3_ (0.207 g, 1.50 mmol) and 3-(bromomethyl)pyridine hydrobromide **5** (0.15 g, 0.60 mmol) and stirred at RT for 12 h. The mixture was diluted with H_2_O (30 ml) and extracted with EtOAc (2 x 40 ml). The combined organic layers were dried (Na_2_SO_4_) and concentrated. Purification of the residue by flash chromatography (SiO_2_ 230-400 mesh, 40% EtOAc in petrol ether) gave 4-[1-(pyridin-3-ylmethyl)benzimidazol-2-yl]-1,2,5-oxadiazol-3-amine C101505 (90 mg, 61%) as an off-white solid1H NMR (400 MHz, DMSO) δ 8.53 (br. d, *J* ≈ 1.8 Hz, 1H), 8.47 (br. dd, *J* ≈ 4.5, 1.3 Hz, 1H), 7.88 (d, *J* = 7.5 Hz, 1H), 7.79 (d, *J* = 7.7 Hz, 1H), 7.51 (br. dt, *J* ≈ 7.9, 1.6 Hz, 1H), 7.37-7.46 (m, 2H), 7.32 (dd, *J* = 7.6, 4.7 Hz, 1H), 7.01 (s, 2H), 6.01 (s, 2H). MS ESI^+^: 293.33 [M + H]^+^. HR-MS (ESI) for C_15_H_13_N_6_O: 293.1145 (calc.), 293.1148 (found). Analyt. UPLC: 99.3%.

The tritiated probe compound **^3^H-**C101505 was prepared in two steps from the benzimidazole intermediate **5** by *N*-alkylation followed by Palladium-catalylzed tritio-debromination.

*4-(1-((6-Bromopyridin-3-yl)methyl)benzimidazol-2-yl)-1,2,5-oxadiazol-3-amine* (**7**). A stirred solution of benzimidazole **3** (0.20 g, 0.91 mmol) in DMF (5 ml) was treated with K_2_CO_3_ (0.378 g, 2.70 mmol) and (6-bromopyridin-3-yl)methyl methanesulfonate **6** (0.172 g, 1.40 mmol) and stirred for 12 h at RT. The mixture was treated with H_2_O and extracted with EtOAc. The organic layer was dried (Na_2_SO_4_) and concentrated under reduced pressure. Purification of the resulting residue by column chromatography (Davisil, 10-30% EtOAc in petrol ether) gave the title compound **7** (0.105 g, 28%) as an off-white solid. ^1^H NMR (400 MHz, DMSO-d_6_): δ 8.38 (d, J = 2.0 Hz, 1H), 7.88 (d, J = 8.0 Hz, 1H), 7.79 (d, J = 8.0 Hz, 1H), 7.57 (d, J = 8.4 Hz, 1H), 7.48-7.39 (m, 3H), 7.00 (s, 2H), 5.97 (s, 2H). MS ESI^+^: 371.35 [M + H]^+^. Analyt. UPLC: 98.9%.

*4-(1-((pyridin-3-yl-6-t)methyl)-1H-benzo[d]imidazol-2-yl)-1,2,5-oxadiazol-3-amine* (^3^H-C101505). A mixture of 2-bromopyridine **7** (2.0 mg, 5.4 μmol) and Pd/CaCO_3_ (5 mg) in DMF (1 ml) was stirred at RT under tritium gas for 0.5 h. The crude material was concentrated *in vacuo* and the residue purified by reverse phase HPLC (Gemini C18 250 x 9.4 mm column; mobile phase A: 0.1% CF_3_COOH in H_2_O, mobile phase B: 0.1% CF_3_COOH in CH_3_CN; 10%- 55% B over 1 h at a flow rate of 3 ml/min). The specific activity of the final product ^3^H-C101505 was 14 Ci/mmol by mass spectrometry. The radiochemical purity as determined by HPLC (XBridge Phenyl 5µm 250 x 4.6 mm column; mobile phase A: 0.1% CF_3_COOH in H_2_O, mobile phase B: 0.1% CF_3_COOH in CH_3_CN; 0-100% B, over 15 min at a flow rate of 1 ml/min) was 99.9%.

**KCNK13 mRNA Generation**

pNeoN8 huKCNK13 (NM_022054.4) was digested with Thermo FastDigest HindIII, at 37°C for 1 hour & denatured at 80°C for 15 mins. Digested DNA precipitated using 1/20th volume 0.5M EDTA, 1/10th volume 3M Sodium Acetate, 2x volume ethanol at -20°C for 50 min. Precipitated DNA was centrifuged at full speed for 15 min at 4°C, supernatant was removed, and pellet washed with 750 µl 70% EtOH, pellet was centrifuged to dry for 5 min, with two further short centrifuge steps for final drying. DNA was resuspended in 20 µl H_2_O. 1 µl was run on 0.7% agarose gel for 30 min at 100 V to confirm linearisation.

mMessage Machine Kit (Invitrogen) was used to generate mRNA from linearised pNeoN8 huTHIK-1 plasmid, as per manufacturers protocol, at 37°C overnight. 1 µl Turbo DNase was added to each reaction and incubated at 37°C for 15 min. PolyA tailing of mRNA was performed as detailed in manufacturer’s protocol for 45 min at 37°C.

MegaClear Purification Kit (Invitrogen) was used to purify mRNA as per manufacturer’s protocol. Once purified, mRNA was freeze dried using a vacuum centrifuge and resuspended in 40 µl H_2_O to achieve a concentration of 4.4 µg/µl. 1 µl mRNA was analysed on an Agilent Bioanalyser RNA Nanochip against untailed mRNA to confirm integrity and tailing.

**Nuclear Enriched Transcript Sort Sequencing (NETSseq)**

Human CNS tissue samples were homogenised in medium (0.25 M sucrose, 150 mM KCl, 5 mM MgCl_2_, 20 mM Tricine pH 7.8, 0.15 mM spermine, 0.5 mM spermidine, 1 mM dithiothreitol (DTT), 20 U/ml Superase-In RNase inhibitor, 40 U/ml RNasin ribonuclease inhibitor, mini EDTA-free protease inhibitor cocktail) that was supplemented with 50% iodixanol solution (50% iodixanol/Optiprep, 150 mM KCl, 5 mM MgCl_2_, 20 mM Tricine pH 7.8, 0.15 mM spermine, 0.5 mM spermidine, mini EDTA-free protease inhibitor cocktail, 1 mM DTT, 20 U/ml Superase-In RNase inhibitor, 40 U/ml RNasin ribonuclease inhibitor) post homogenisation, and laid on a 27% iodixanol cushion. Nuclei were pelleted by centrifugation 25 min, 10,000×g at 4°C (Eppendorf 5427 R centrifuge, FA-45-12-17 rotor).

| **Kinase** | **IC_50_ (μM)** |
| --- | --- |
| p70S6K (h) | >10.00 |
| PKA (h) | >10.00 |
| GSK3β (h) | >10.00 |
| ROCK-1 (h) | >10.00 |
| ROCK-2 (h) | >10.00 |
| Lck (h) | >10.00 |
| Syk (h) | >10.00 |

**Supplementary Table 1**: kinase selectivity data for C101248

| Target | Species | % inhibition at 10 µM (n=2) |
| --- | --- | --- |
| Phosphodiesterase PDE3 | Human | 1 |
| Adrenergic α1, Non-Selective | Rat | 12 |
| Adrenergic β2 | Human | 15 |
| Androgen (Testosterone) | Human | 6 |
| Calcium Channel CaV1.2 | Human | 12 |
| Calcium Channel L-Type, Benzothiazepine | Rat | 3 |
| Cannabinoid CB1 | Human | 22 |
| Cannabinoid CB2 | Human | 0 |
| Dopamine D1 | Human | -1 |
| GABAA, Flunitrazepam, Central | Rat | -9 |
| Histamine H1 | Human | 21 |
| Muscarinic M1 | Human | -3 |
| Opiate μ (OP3, MOP) | Human | -9 |
| Serotonin (5-Hydroxytryptamine) 5-HT2B | Human | 24 |
| Sodium Channel, Site 2 | Rat | 2 |
| Transporter, Dopamine (DAT) | Human | 91 |
| Transporter, Norepinephrine (NET) | Human | 23 |
| Transporter, Serotonin (5-Hydroxytryptamine) (SERT) | Human | -7 |

**Supplementary Table 2**: Promiscuity screening panel data for C101248. Compound was screened at 10 µM, in duplicate, using radiometric binding assays

**
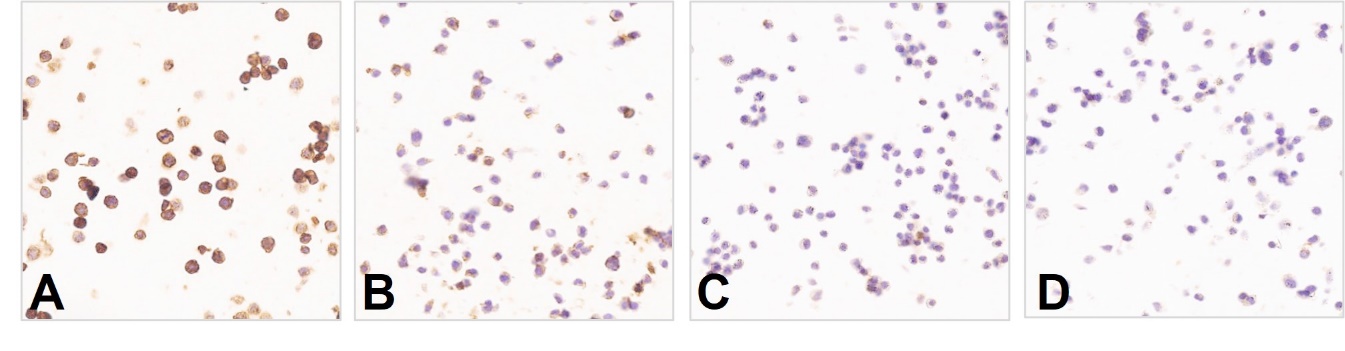
**

**Supplementary Figure 1.** Digital scans (20x Aperio Scanscope) showing experimental validation of anti-THIK-1 antibody in HEK-hTHIK cells (A and C) and parental HEK293 cells (B and D). Cells in A and B were incubated with an anti-THIK-1 antibody and those in C and D with non-immune rabbit IgG under identical immunocytochemical conditions employing tyramide amplification. Very low levels of non-specific staining were present in C and D following negative control incubations, while clear differences in the levels of immunoreactivity can be seen in HEK-hTHIK-1 and parental cells.

**Supplementary Figure 2.** TPA inhibition of thallium influx in HEK-hTHIK-1 and HEK-mTHIK-1 cells. The data shown is the averaged responses (± SD) from 2 independent experiments, each performed in duplicate.


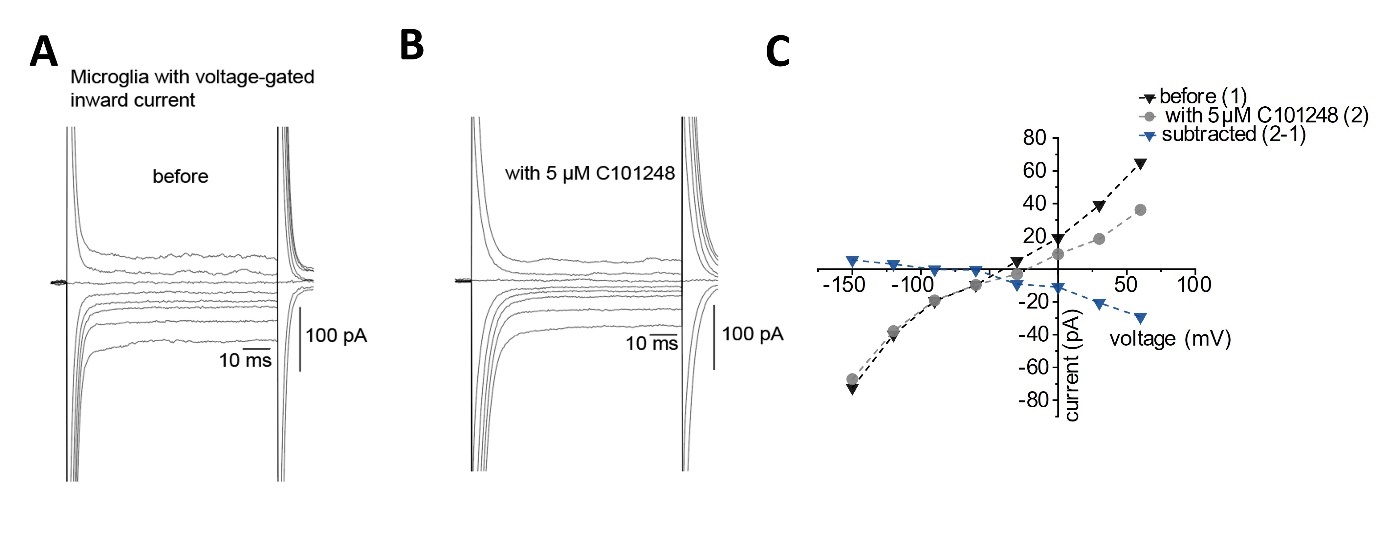


**Supplementary Figure 3:** Inward rectifier K^+^ channels are unaffected by C101248. Specimen recordings showing the voltage-dependent current pattern in microglia in response to depolarizing voltage steps ranging from -150 mV to +60 mV with 30 mV increments from a holding potential of 0 mV in the absence (A) and presence (B) of 5 µM C101248. The current-voltage relationship (C) shows the block of the constitutive current component of the outwardly rectifying potassium channel THIK-1 (inverted net current, blue line), whereas voltage-gated inward currents, mainly reflecting inwardly-rectifying K+ currents, are not affected by C101248.

**
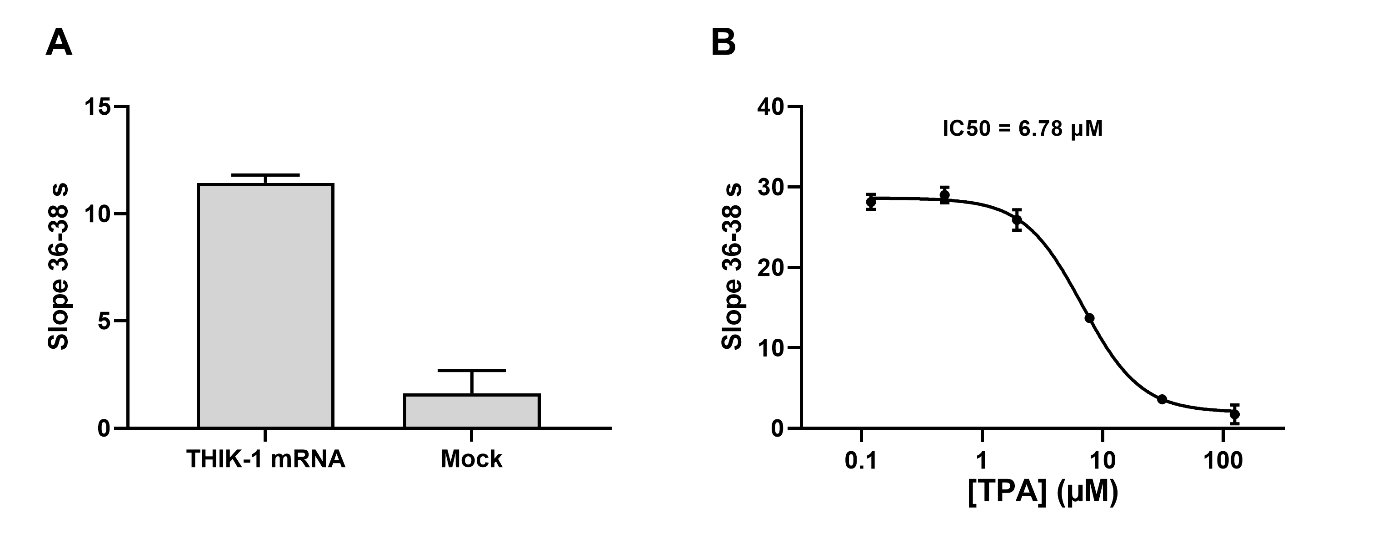
**

**Supplementary Figure 4**. Thallium influx in human THIK-1 overexpressing differentiated THP1 cells. Comparison of THIK-1 mRNA vs mock transfected cells (A). Data represent the mean ± SD from two independent experiments. Concentration-dependent inhibition of thallium influx in THIK-1 mRNA-transfected cells (B). Data represent the mean ± SD from two independent experiments carried.
